# Ubiquitination and deubiquitination in cancer: from mechanisms to novel therapeutic approaches

**DOI:** 10.1186/s12943-024-02046-3

**Published:** 2024-07-25

**Authors:** Fangfang Liu, Jingyu Chen, Kai Li, Haochen Li, Yiyi Zhu, Yubo Zhai, Bingbing Lu, Yanle Fan, Ziyue Liu, Xiaojie Chen, Xuechao Jia, Zigang Dong, Kangdong Liu

**Affiliations:** 1 Tianjian Laboratory of Advanced Biomedical Sciences, Academy of Medical Sciences, College of Medicine, Zhengzhou University, Zhengzhou, Henan 450001 China; 2 China-US (Henan) Hormel Cancer Institute, Zhengzhou, Henan 450000 China; 3 Department of Pediatric Medicine, School of Third Clinical Medical Sciences, Zhengzhou University, Zhengzhou, 450000 China; 4 Department of Clinical Medicine, School of First Clinical Medical Sciences, Zhengzhou University, Zhengzhou, 450000 China; 5 Department of Pathophysiology, School of Basic Medical Sciences, Zhengzhou University, Zhengzhou, 450000 China; 6 School of Basic Medicine, Henan University of Science and Technology, Luoyang, China; 7 Henan International Joint Laboratory of TCM Syndrome and Prescription in Signaling, Traditional Chinese Medicine (Zhong Jing) School, Henan University of Chinese Medicine, Zhengzhou, Henan China

**Keywords:** Ubiquitination, Cancer hallmarks, Molecular mechanisms, Targeted therapies, Immunotherapies

## Abstract

Ubiquitination, a pivotal posttranslational modification of proteins, plays a fundamental role in regulating protein stability. The dysregulation of ubiquitinating and deubiquitinating enzymes is a common feature in various cancers, underscoring the imperative to investigate ubiquitin ligases and deubiquitinases (DUBs) for insights into oncogenic processes and the development of therapeutic interventions. In this review, we discuss the contributions of the ubiquitin–proteasome system (UPS) in all hallmarks of cancer and progress in drug discovery. We delve into the multiple functions of the UPS in oncology, including its regulation of multiple cancer-associated pathways, its role in metabolic reprogramming, its engagement with tumor immune responses, its function in phenotypic plasticity and polymorphic microbiomes, and other essential cellular functions. Furthermore, we provide a comprehensive overview of novel anticancer strategies that leverage the UPS, including the development and application of proteolysis targeting chimeras (PROTACs) and molecular glues.

## Introduction

**Fig. 1:**
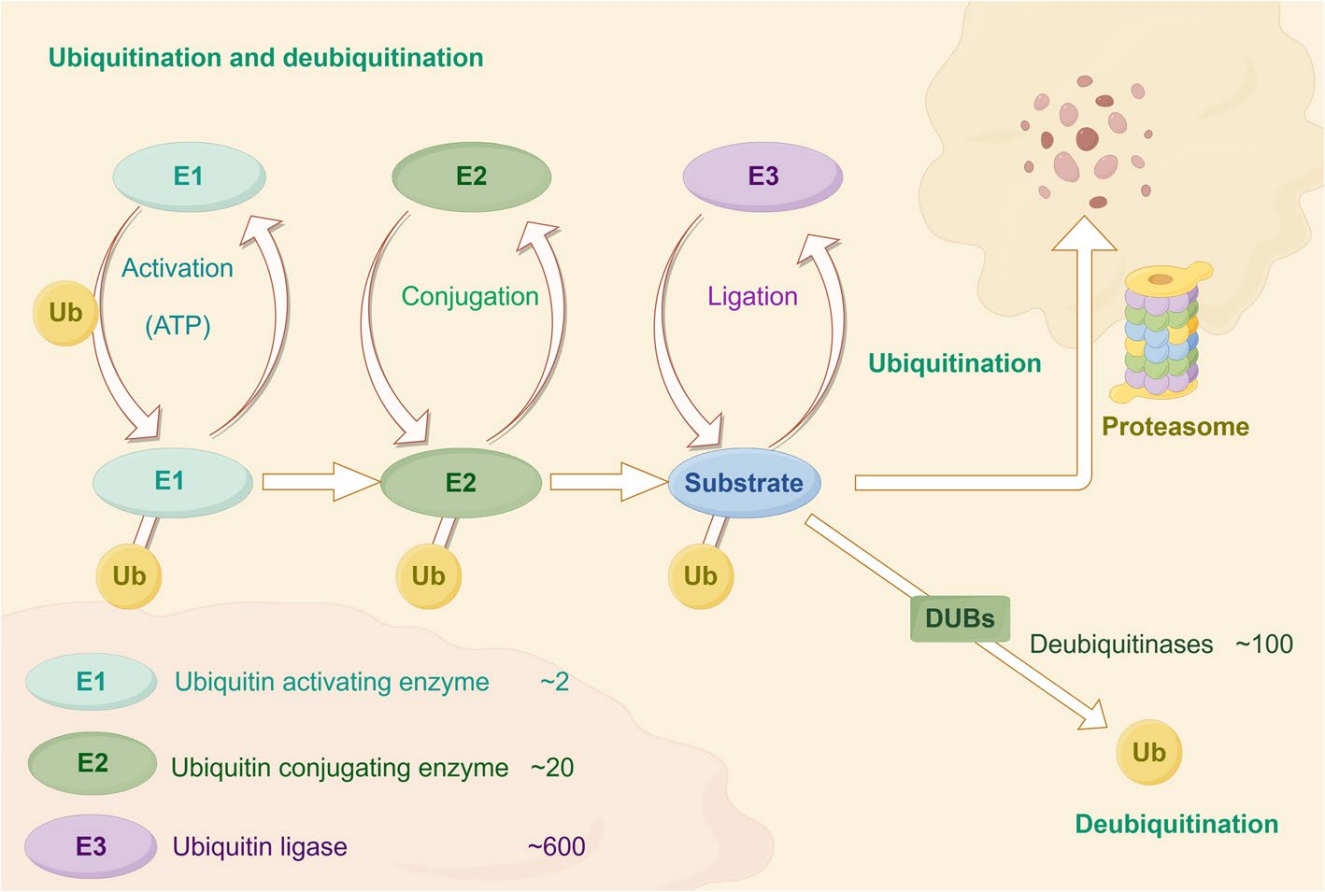
The processes of ubiquitination and deubiquitination occur within the ubiquitin–proteasome system (UPS). Ubiquitination involves the sequential action of three enzyme classes: ubiquitin-activating enzymes (E1), ubiquitin-conjugating enzymes (E2), and ubiquitin-protein ligases (E3). Initially, E1 enzymes activate ubiquitin (Ub) in an ATP-dependent process. Subsequently, the activated Ub is transferred to E2 enzymes via a thioester bond. The final step is catalyzed by E3 ligases, which facilitate the transfer of Ub from E2 to the target substrate protein, marking it for degradation

Ubiquitin (Ub) is composed of 76 amino acids and is found in all eukaryotic tissues [1]. Ubiquitination is the second most common posttranslational modification of proteins following phosphorylation [2]. Ubiquitination is a highly specific process of ATP-dependent cascade labeling substrate proteins with ubiquitin [3]. Moreover, ubiquitin and its degradation by the proteasome constitute the ubiquitin–proteasome system (UPS), which is responsible for 80–90% of cellular proteolysis and 10–20% of autophagy [4]. The ubiquitination modification involves a series of reactions mediated by a ubiquitin activating enzyme (E1), a ubiquitin conjugating enzyme (E2), and a ubiquitin ligase (E3) (Fig. 1) [5]. Ubiquitination plays a crucial regulatory role in the modulation of tumors, impacting cellular survival, proliferation, and differentiation. At the same time, ubiquitination is reversible, and ubiquitin or ubiquitin chains linked to substrate proteins can be removed by deubiquitinases (DUBs).

The emerging functions of ubiquitination and deubiquitination in regulating cancer hallmarks, including “evading growth suppressors,” “reprogramming energy metabolism,” “unlocking phenotypic plasticity,” “polymorphic microbiomes,” and “senescent cells,” have been revealed [6, 7]. The UPS can regulate the protein levels of programmed cell death 1/programmed cell death ligand 1 (PD-1/PD-L1) in the tumor microenvironment (TME) and enhance the effectiveness of immunotherapy [8]. For example, ubiquitin-specific protease 2 (USP2), a DUB, can stabilize PD-1 and promote tumor immune escape through deubiquitination [9]. Moreover, the UPS also regulates tumor metabolic reprogramming. Recent investigations have revealed that the E3 ligase Parkin can facilitate the ubiquitination of pyruvate kinase M2 (PKM2) [10]. In addition, the DUB OTU domain-containing ubiquitin aldehyde-binding protein 2 (OTUB2) interacts with PKM2 to inhibit PKM2 ubiquitination by the E3 ligase Parkin, enhancing glycolysis and accelerating colorectal cancer progression [11]. Many new reports have recently revealed novel ubiquitination methods for cancer treatment, such as PROTACs and molecular glues. PROTAC technology is a valuable platform for driving the degradation of target proteins. ARV-110 (alias bavdegalutamide) and ARV-471 (alias vepdegestrant) represent the forefront of PROTAC drug development in clinical trials and have progressed to phase II trials [12]. Compared to PROTACs, molecular glues have smaller molecular dimensions, simplifying the optimization of their chemical characteristics. A few molecular glue degradants have been identified. Notably, CC-90009 facilitates the ubiquitination-mediated degradation of G1-to-S phase transition 1 (GSPT1) by recruiting the E3 ligase complex CUL4-DDB1-CRBN-RBX1 (CRL4^CRBN^). It is in phase II clinical trials for leukemia therapy [13]. ARV-110 is designed to selectively target and bind to the androgen receptor (AR), facilitating its degradation by recruiting an E3 ubiquitin ligase. Early data from the first-in-human phase I study revealed the safety and tolerability of ARV-110 in patients diagnosed with metastatic castration-resistant prostate cancer [14]. Our research team recently identified a drug that promotes protein ubiquitination and degradation. Indomethacin, for instance, diminishes the growth and recurrence of esophageal squamous cell carcinoma (ESCC) by enhancing the E3 ligase synovial apoptosis inhibitor 1 (SYVN1)-mediated ubiquitination of integrin αv (ITGAV) [15]. We also discovered that honokiol directly interacts with keratin 18 (KRT18), inhibiting melanoma growth by inducing KRT18 ubiquitination and degradation [16].

In this review, we integrate the ubiquitination and deubiquitination processes with the 14 hallmarks of cancer. We clarify the fundamental mechanisms and functions of ubiquitination and deubiquitination in tumor suppression, metabolic reprogramming, immune evasion, phenotypic plasticity, polymorphic microbiomes, and other essential cellular functions, focusing on recent developments. Finally, we explore the therapeutic potential of targeting the UPS in cancer therapy.

## Functions of various types of ubiquitination in cancer

**Fig. 2:**
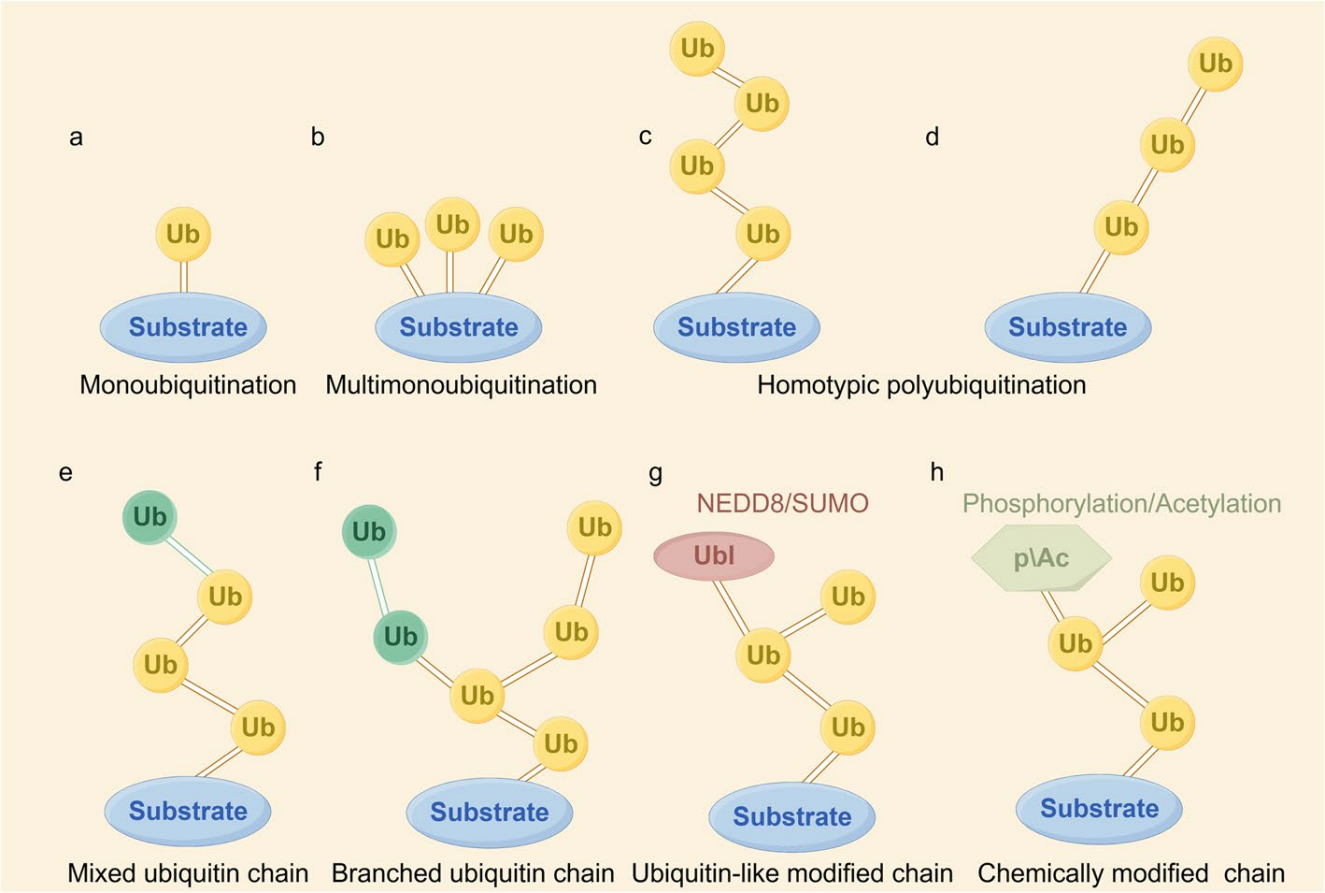
The various types of ubiquitin (Ub) linkages are as follows. **a** Mono-ubiquitination: A single ubiquitin protein is attached to a substrate protein. **b** Multi-monoubiquitination: Multiple ubiquitin proteins are each linked to different sites on the same substrate protein. **c** Homotypic polyubiquitination: Ubiquitin can bind to another ubiquitin through one of its seven lysine residues (K6, K11, K27, K29, K33, K48, and K63) or the N-terminal methionine residue (M1). Multiple identical ubiquitin proteins form a chain, which is then attached to a substrate protein. **d** Linear ubiquitination: A specific form of homotypic polyubiquitination where ubiquitin molecules are connected via Met1 linkages. **e** Mixed ubiquitin chain: A ubiquitin can be linked by two or more different connection methods within the same polymerization reaction, resulting in mixed ubiquitin chains. **f** Branched ubiquitin chain: Ubiquitin proteins in a chain are modified by adding more ubiquitin proteins at different binding sites. **g** Ubiquitin-like modified chain: A ubiquitin protein in the chain is linked to a ubiquitin-like protein. **h** Chemically modified chain: Ubiquitin proteins in the chain are modified by other protein modifications, such as phosphorylation or acetylation. The formation of mixed ubiquitin chains, branched ubiquitin chains, ubiquitin-like modified chains, and chemically modified ubiquitin chains are collectively referred to as heterotypic polyubiquitination

Ubiquitination can be categorized into monoubiquitination, multimonoubiquitination, homotypic polyubiquitination, and heterotypic polyubiquitination (Fig. 2) [4]. Monoubiquitination refers to the attachment of a single ubiquitin protein to a substrate protein. When multiple ubiquitin proteins are attached to different lysine residues on the same substrate protein, it is termed multimonoubiquitination. Ubiquitin itself contains eight potential linkage sites, which include seven lysine residues (K6, K11, K27, K29, K33, K48, and K63) and one N-terminal methionine residue (M1) [17]. These sites allow for the formation of polyubiquitin chains through further ubiquitin attachment. Homotypic polyubiquitination occurs when ubiquitin proteins are linked through the same residue type, creating a uniform chain [4]. In contrast, heterotypic polyubiquitination involves ubiquitin proteins linked through different residue types, resulting in mixed or branched chains [18]. When more than one ubiquitin molecule is simultaneously linked to a single ubiquitin molecule, the resulting ubiquitin chain is referred to as a branched ubiquitin chain [18]. A family of proteins, known as ubiquitin-like proteins (UBLs), shares structural and functional characteristics with ubiquitin, playing similar roles in modulating protein activity and cellular processes. This family encompasses proteins such as neural precursor cell-expressed developmentally downregulated 8 (NEDD8), small ubiquitin-related modifier (SUMO), and interferon-stimulated gene 15 (ISG15) [17]. A ubiquitin-like modified chain means that the substrate or ubiquitin is modified by a ubiquitin-like protein. In addition, ubiquitin can also be posttranslationally modified through phosphorylation and acetylation, which is called a chemically modified ubiquitin chain. The formation of mixed ubiquitin chains, branched ubiquitin chains, ubiquitin-like modified chains, and chemically modified ubiquitin chains are collectively referred to as heterotypic polyubiquitination [4].

### Monoubiquitination

Previous studies have shown that monoubiquitination of proteins acts as a signal for DNA repair, signal transduction, and phagocytosis in vivo [19, 20]. Recently, histone monoubiquitination has been widely studied. Histone monoubiquitination often occurs on H2A and H2B. Ring finger protein 2 (RNF2) is an E3 ligase with a RING domain [21]. RNF2 facilitates the monoubiquitination of histone H2A at lysine 119, leading to the recruitment of E-cadherin to the promoter region and subsequent transcriptional repression of E-cadherin. This mechanism contributes to enhancing the metastatic potential of hepatocellular carcinoma [22]. In addition, ubiquitin-conjugating enzyme E2T (UBE2T) regulates the monoubiquitination of the histone variant H2AX (γH2AX). This process induces the phosphorylation of cell cycle checkpoint kinase 1 (CHK1), thereby enhancing the radioresistance of hepatocellular carcinoma cells [23]. Monoubiquitination also plays an essential role in immune escape. Metastasis suppressor protein 1 (MTSS1) promotes the monoubiquitination of the immune checkpoint PD-L1 at K263 mediated by the E3 ligase atrophin-interacting protein 4 (AIP4), which leads to the internalization of PD-L1, endosomal transport, and lysosomal degradation, thus inhibiting the immune escape of lung adenocarcinoma [24]. Additionally, the ubiquitin-binding enzyme E2B (UBE2B) can facilitate the monoubiquitination of the transcription regulator zinc finger MYM-type protein 2 (ZMYM2) mediated by the ubiquitin E3 ligase ring finger protein 73 (RNF73), thereby promoting the growth of ovarian cancer [25]. These observations suggest that the monoubiquitination of proteins primarily affects the growth, metastasis, radiation resistance, and immune escape of cancer cells by affecting DNA repair and gene transcription.

### Linear ubiquitination

The ubiquitin chains assembled by M1 are called linear ubiquitin chains. These chains are assembled exclusively by the E3 ligase linear ubiquitin chain assembly complex (LUBAC) and are disassembled by OTU deubiquitinase with linear linkage specificity (OTULIN) and cylindromatosis (CYLD) [26, 27]. LUBAC consists of HOIL-1 interacting protein (HOIP), heme-oxidized IRP2 ubiquitin ligase 1 (HOIL-1L), and SHANK-associated RH domain-interacting protein (SHARPIN) [28]. The impact of linear ubiquitin chains on cancer has been extensively investigated. Met1-Ub signaling plays a vital role in many aspects of cancer through NF-κB regulation. HOIP promotes lymphoma by activating NF-κB signal transduction, indicating that LUBAC is a viable therapeutic target for B-cell lymphoma [29]. Epsin, a member of the ubiquitin-binding endocytosis adaptor protein family, engages with the linear ubiquitin chain assembly complex (LUBAC) to facilitate the linear ubiquitination of the NF-κB essential modulator (NEMO). This interaction is implicated in the progression of breast cancer [30]. Protein kinase transforming growth factor β-activated kinase 1 (TAK1) is the main mediator of NF-κB activation in the LUBAC-dependent mechanism. Targeting LUBAC or TAK1 may be an attractive therapeutic strategy for A20-mutant Hodgkin’s lymphoma [31]. RANBP2-type and C3HC4-type zinc finger containing 1 (RBCK1) was first identified as an essential component of LUBAC and promoted NF-κB signal transduction during the immune response [32]. Furthermore, the phosphorylation of OTULIN facilitates the activation of the genotoxic Wnt/β-catenin pathway, thereby augmenting drug resistance in breast cancer [28]. Consequently, the Met1-linked linear ubiquitin chain acts as an essential positive modulator of NF-κB signaling pathways, playing pivotal roles in oncogenesis, inflammation, and immune regulation.

### K48-linked polyubiquitination

K48-linked polyubiquitination is the most widely studied type and the main connection type in cells. It mainly marks proteins that are recognized and degraded by the 26S proteasome and targets proteins for proteasomal degradation [18]. The E3 ligase tripartite motif protein 7 (TRIM7) can directly interact with the tyrosine kinase Src, induce the ubiquitination of Lys48-linked Src, reduce the abundance of the Src protein in hepatocellular carcinoma cells, and inhibit the progression of hepatocellular carcinoma [33]. Recently, a new circRNA involved in hypoxia reactions named circular insulin-induced gene 1 (circINSIG1) was identified. CircINSIG1 encodes the protein circINSIG1-121, which has 121 amino acids. By recruiting the E3 ligase CUL5-ASB6 complex, circINSIG1-121 promotes the ubiquitination of the critical cholesterol metabolism regulator INSIG1 at the K48 linkage of lysine 156 and lysine 158, thus inducing cholesterol biosynthesis and promoting colorectal cancer proliferation and metastasis [34]. In addition, the E3 ligase MG53 catalyzes the K48-linked ubiquitination and subsequent degradation of cyclin D1, thus inhibiting the growth of colorectal cancer [35]. However, in the ubiquitinating enzyme family, studies have shown that methyltransferase 5, N6-adenosine (METTL5) regulates the translation of USP5 and suppresses K48-linked ubiquitination of c-Myc, thus reprogramming glucose metabolism and promoting the progression of hepatocellular carcinoma [36]. Therefore, as the most widely studied ubiquitination form, K48-linked polyubiquitination plays a key role in various aspects of cancer by promoting the degradation of corresponding proteins.

### K63-linked polyubiquitination

K63-linked polyubiquitination participates in signal assembly and promotes the autophagic degradation of protein substrates. It can also regulate nondegradative processes, such as protein transport, DNA repair, and protein kinase activation [37]. The AB22A-NeoF1 fusion gene encodes the Rab22a-NeoF1 fusion protein, which coordinates various mechanisms to facilitate lung metastasis in osteosarcoma [38]. The E3 ligase STIP1 homology and U-box-containing protein 1 (STUB1) catalyzes the K63-linked ubiquitination of K112 of the Rab22a-NeoF1 fusion protein, which promotes the lung metastasis of osteosarcoma [39]. K63-linked polyubiquitination also plays an important role in immune escape. For instance, the E3 ligase TRIM28 promotes the K63-linked ubiquitination of TANK-binding kinase 1 (TBK1). It activates the TBK1-IRF1 and TBK1-mTOR pathways, thus enhancing the transcription of PD-L1 and promoting the escape of gastric cancer cells from immune surveillance [40]. In addition, mind bomb homolog 2 (MIB2) catalyzes the ubiquitination of PD-L1 at the K63 linkage, but not its degradation, and promotes tumor immune escape [41]. In addition, anillin (ANLN) is a mitotic protein that can promote the formation of contractile rings and cell division. The results showed that USP10 removes the K11- and K63-linked ubiquitin chains of ANLN through its ubiquitinating enzyme activity and prevents the ubiquitin-mediated degradation of ANLN, effectively inhibiting the cell cycle procession of ESCC [42]. Taken together, K63-linked polyubiquitination plays an important role in cancer metastasis, immune escape, and the cell cycle.

### Other types of polyubiquitination

Relatively few modified substrates and functions of “atypical” ubiquitin chains (K6, K11, K27, K29, K33, and M1 chains) are known [43, 44]. The ubiquitination of K11 is mainly related to UBE2S. Previous research revealed that UBE2S stabilizes β-catenin via K11-linked ubiquitination, contributing to the development of colorectal cancer [45]. In addition, UBE2S interacts with TRIM21, which degrades lipoma preferred partner (LPP) through ubiquitination linked with K11 and promotes the lymphatic metastasis of bladder cancer [46]. The K29-linked ubiquitin chain plays a significant role in driving cancer invasion and metastasis and in the positive regulation of immunity [47]. Recent studies have demonstrated that ring finger protein 167 (RNF167) activates mTORC1 and promotes the occurrence of breast cancer by targeting and degrading K29-linked ubiquitinated cytosolic arginine sensor for mTORC1 subunit 1 (CASTOR1). In addition, this observation confirmed that RNF167 is a therapeutic target of breast cancer [48].

In addition to eight homotypic polyubiquitination modifications, heterotypic polyubiquitination modifications also occur widely in cells [18]. These modifications predominantly involve mixed and branched polyubiquitination, characterized by the formation of polyubiquitin chains on substrates that feature two distinct types of lysine linkages, resulting in complex ubiquitin chain configurations [49]. Poly(A)-binding protein, cytoplasmic 1 (PABPC1), is an extensively studied protein, and recent research has revealed its involvement in the tumorigenesis of numerous cancers. CDC2-like kinase 2 (CLK2) is a bispecific kinase, that facilitates the phosphorylation of diverse proteins, and an increasing amount of data indicate that CLK2 functions as an oncogenic kinase [50]. USP10 can reverse K27/29-linked ubiquitination of PABPC1 and upregulate the translation of CLK2, thus promoting tumor development of pancreatic ductal adenocarcinoma (PDAC) [51].

## Ubiquitination and deubiquitination regulate the hallmarks of cancer

### Sustained proliferative signaling

**Fig. 3:**
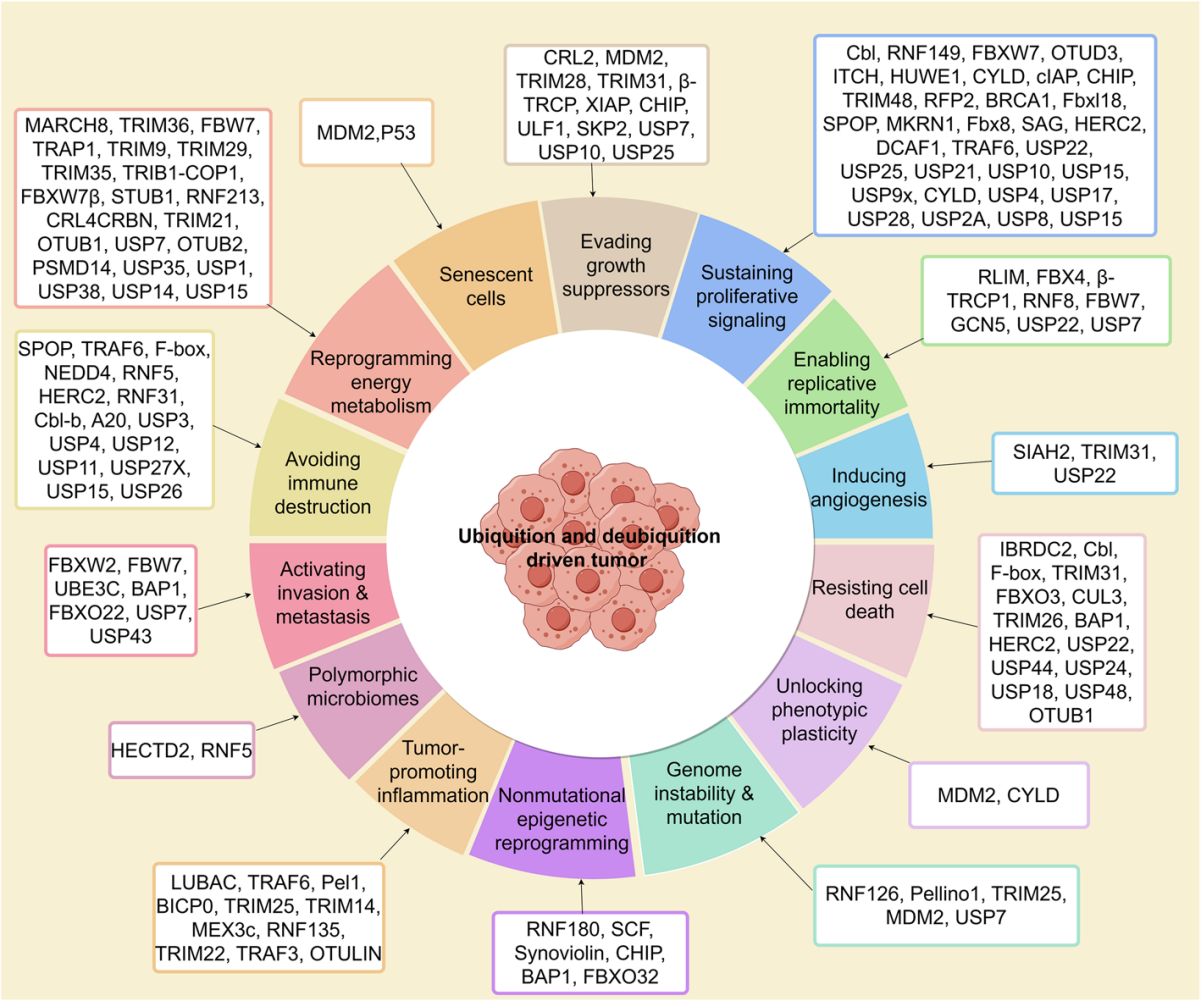
Ubiquitination and deubiquitination regulation of the hallmarks of cancer. E3 ubiquitin ligases and deubiquitinating enzymes, by regulating the degradation and stability of proteins, significantly influence the hallmarks of malignant tumors, which include sustained proliferative signaling, evading growth suppressors, resisting cell death, enabling replicative immortality, inducing angiogenesis, activating invasion and metastasis, genome instability and mutation, tumor-promoting inflammation, reprogramming energy metabolism, evading immune destruction, unlocking phenotypic plasticity, nonmutational epigenetic reprogramming, polymorphic microbiomes, and senescent cells. Each cancer hallmark-associated E3 ligase and deubiquitinating enzyme (DUB) is listed in the corresponding box

The ability to maintain cancer cell proliferation is a fundamental characteristic of cancer cells. Normal cells can control the production and release of growth-promoting signals. However, cancer cells can escape the control of these signals and obtain sustained proliferative stimulation (Fig. 3) [6].

#### Epidermal growth factor receptor (EGFR) signaling pathway

The EGFR-dependent signaling pathway maintains cell proliferation, and its dysregulation increases cancer cell proliferation [52, 53]. EGFRvIII is the most common mutation in gliomas [54]. Tumors with EGFRvIII mutations exhibit an increase in phosphorylation at Y371 of casitas B-lineage lymphoma (Cbl), a critical residue regulating E3 activity. The conformational changes in Cbl are essential for EGFR ubiquitination in vitro. EGFRvIII mutations can reduce EGFR protein degradation in tumors by inhibiting Cbl activation (Table 1) [55, 56]. It was indicated that β-Element, a traditional Chinese herb, played an anti-tumor and anti-metastatic role in multidrug-resistant (MDR) gastric cancer by suppressing EGFR levels through Cbl-b upregulation [57]. Additionally, a high expression level of Cbl-b is significantly associated with improved prognosis in patients with lung adenocarcinoma, suggesting its potential as a prognostic biomarker for better clinical outcomes [58]. Recent studies have reported that the overexpression of DUBs such as Cezanne-1, USP22, and USP25 in cancer cells prevents EGFR degradation [59–61]. In clinical studies, Cezanne-1 is often amplified in tumor samples from breast cancer patients, with elevated levels of its mRNA associated with a poor prognosis [62]. Similarly, there is a significant association between USP22 levels and poor prognosis in neuroblastoma [63]. Notably, an in vivo study suggested that USP25/28 inhibitor showed a potent anti-tumor effect on pancreatic cell-derived xenograft (CDX) mouse model, implying combining DUB inhibitors with EGFR inhibitors or chemotherapeutic agents may enhance therapeutic efficacy [64]. In conclusion, these observations suggest that E3 ligases and DUBs regulate EGFR signaling, opening new avenues for targeted therapeutic strategies against cancer cell proliferation.

**Table 1:** E2 enzymes, E3 ligases, and DUBs in the regulation process of cancer hallmarks

Cancer hallmarks	E2s/E3s/DUBs	Name	Substrate	Cancer type	Outcomes	References
1.Sustaining proliferative signaling	E3s	RNF7	PI3K/AKT signaling pathway	Pancreatic cancer	Activates the PI3K/AKT signaling pathway and promotes tumor formation	[65]
		RNF41	HER3	Breast cancer	Degrades HER3 and promotes tumor proliferation	[66]
		MARCH6	DHX9	Primary papillary thyroid cancers	Activates AKT/mTOR signaling pathway and promotes tumor proliferation and metastasis	[67]
		ANKRD9	IMPDH	Gastric cancer	Promotes ubiquitination and proteasomal degradation of IMPDH to suppress tumor growth	[68]
		Cbl	EGFR	Lung cancer	Promotes tumor growth	[55, 56]
		MKRN1	PTEN	Cervical cancer	Ubiquitinates PTEN protein and promotes cancer proliferation	[69]
		BCR-ABL	SHIP	Hematopoietic tumors	Promotes SHIP proteasomal degradation	[70]
		FBX8	mTOR	Colorectal cancer	Targets mTOR for degradation	[71]
		SAG	PHLPP1/DEPTOR	Prostate cancer	Inactivates the PI3K/AKT/mTOR axis	[72]
		MULAN	AKT	Head and neck cancer	Promotes the ubiquitination and degradation of AKT1 and AKT2	[73]
		RFP2	AKT/MDM2	Multiple myeloma	Degrades AKT and MDM2	[74]
		BRCA1	AKT	Breast cancer	Ubiquitinates and directly degrades AKT1	[75]
		CHIP	AKT	Cervical Cancer	Ubiquitinates and directly degrades ASK1	[76]
		SKP2	AKT	Breast cancer	Promotes tumor occurrence and metastasis	[77]
		TRAF4	AKT	Lung cancer	Promotes tumorigenesis	[78]
		FBXL18	AKT	Glioma	Promotes tumor proliferation and development	[79]
	DUBs	USP17/USP4	PDFGRβ	Osteosarcoma	Promotes aberrant STAT3 transcription	[80]
		USP15	ERα	Breast cancer	Blocks the ubiquitination and degradation of ERα	[81]
		USP4	TAK1	Esophageal squamous cell carcinoma	Stabilizes the TAK1 protein level	[82]
		USP7	ERα	Breast cancer	Deubiquitinates ERα and promotes tumor proliferation	[83]
		Ataxin-3	PTEN	Lung cancer	Restricts PTEN transcription	[84]
		USP13	PTEN	Breast cancer	Deubiquitinates PTEN	[85]
		USP10	PTEN	Lung cancer/ hepatocellular carcinoma	Upregulates PTEN	[86]
		USP10	PTEN	Lung cancer	Inhibits lung cancer cell growth and invasion by upregulating PTEN	[87]
		OTUD3	PTEN	Breast cancer	Upregulates PTEN and suppresses tumorigenesis	[88]
		USP46	PHLPP1	Colon cancer	Functions as a tumor suppressor by controlling PHLPP-dependent attenuation of AKT signaling	[89]
		USP1	PHLPP1	Lung cancer	Regulates AKT phosphorylation by modulating the stability of PHLPP1	[90]
		USP21	MEK2	Hepatocellular carcinoma	Maintains MEK2 stability and activates ERK signaling	[91]
		USP12/WRD48	PHLPP1	Colon cancer	Suppresses AKT-dependent cell survival signaling by stabilizing PHLPP1	[92]
		USP12/UAF-1/WDR20	PHLPP1	Prostate cancer	Regulates the interaction between the androgen receptor and the AKT pathway	[93]
2. Evading growth suppressors	E3s	MDM2	p53	Multiple cancers	Promotes tumor development	[94]
		TRIM28	p53	Lung cancer	Promotes tumor development	[95]
		TRIM31	p53	Pancreatic cancer/Lung cancer	Promotes tumor development	[96]
		TRIM28	RB	Multiple cancers	Promotes tumor development	[95]
		CRL2	ARF	Multiple cancers	Promotes tumor development	[97]
		ULF1	ARF	Hepatocarcinoma	Promotes tumor development	[98]
		Β-TRCP	β-catenin	Multiple cancers	Promotes tumor development	[99]
		F-box	Β-catenin	Colorectal cancer	Promotes tumor development	[100]
		SKP2	NBS1	Prostate cancer	Promotes tumor development	[101]
		CUL1	CHK2	Breast cancer/Ovarian cancer	Promotes DDR	[102]
	DUBs	USP10	p53 /ARF	Multiple cancers	Inhibits tumor development	[98, 103]
		USP7	p53	Multiple cancers	Inhibits tumor development	[104]
3. Resisting cell death	E3s	IBRDC2	BAX	Colorectal cancer	Promotes apoptosis	[105, 106]
		Cbl	BimEL	Multiple cancers	Inhibits apoptosis	[105]
		Peli1	RIPK1	Breast cancer/Lung cancer/Lymphoma	Promotes apoptosis	[107]
		TRIM31	NLRP3	Colitis-associated cancer	Promotes NLRP3 inflammasome	[96, 108]
		CUL3	BECN1	Breast cancer/Ovarian cancer	Promotes autophagy Promotes tumor development	[109]
		TRIM26	SLC7A11	Liver cancer	Promotes tumor development	[110]
	DUBs	A20	RIPK3	Colorectal cancer	Promotes necroptosis	[105, 111]
		USP22	RIPK3	Colorectal cancer	Promotes necroptosis	[105, 111]
		USP24	GSDMB	Bladder cancer	Inhibits pyroptosis	[112]
		USP48	GSDME	Pancreatic cancer	Promotes pyroptosis	[113]
		OTUB1	GPX4	Gastric cancer	Inhibits ferroptosis	[114]
4. Enabling replicative immortality	E3s	Rlim	TRF1	Renal cell carcinoma	Binds to the region between TRFl dimerization and Myb domains to promote tumor growth	[115]
		FBX4	TRF1	Lung cancer	Binds to the N-terminal region of the TRFH dimerization domain of free TRF1 to promote tumor growth	[116]
		β-TrCP1	TRF1	Leukemia	Ubiquitinates TRF1 and promotes tumor growth	[117]
		FBW7	TPP1	Lung cancer	Ubiquitinates TPP1 and promotes tumor growth	[118]
		SIAH1	TRF2	Colorectal cancer	Targets TRF2 degradation promotes tumor proliferation	[119]
	DUBs	USP7	TPP1	Lung cancer	Promotes tumor growth	[120]
5. Inducing angiogenesis	E3	SIAH2	NRF-1	Breast cancer	Decreased activity of SIAH2 and promotes cancer development	[121]
6. Activating invasion and metastasis	E3s	FBXW2	SKP2/β-catenin	Prostate cancer/Lung cancer/Hepatocellular carcinoma Non-small cell lung cancer/Gastric cancer/Ovarian cancer	Promotes ubiquitination and degradation of oncogenic proteins and inhibits tumor migration, invasion, and metastasis	[122–125]
		FBW7	Brg1		Ubiquitinates a variety of oncogenic proteins	[126–130]
		UBE3C	AHNAK	Breast cancer/Hepatocellular carcinoma/Renal cell carcinoma	Promotes tumor invasion and metastasis	[131–133]
		FBXO22	PTEN	Colorectal cancer/Hepatoma	Promotes tumor invasion and metastasis	[134, 135]
	DUBs	BAP1	PTEN	Prostate cancer/Intrahepatic cholangiocarcinoma	Promotes tumor proliferation	[136, 137]
		USP7	EZH2	Prostate cancer/Breast cancer	Enhances the stability of FOXA 1 protein and promotes tumor proliferation	[138–140]
		USP43	NUDR	Breast cancer	Promotes tumor invasion and metastasis	[141]
7. Genome instability and mutation	E3s	RNF126	MRE11	Triple-negative breast cancer	Confers resistance of triple-negative breast cancer to radiotherapy	[142]
		TRIM25	Ku80	Esophageal cancer/Pancreatic cancer	Intensifies DNA damage	[143]
		MDM2	DICER	Breast cancer	Impairs DDR and promotes cancer progression	[144]
	DUBs	USP44	TRIM25	Nasopharyngeal carcinoma	Intensifies DNA damage	[143]
		USP7	SAMHD1	Colonic adenocarcinoma/Lung adenocarcinoma/Glioblastoma/Glioma	Repairs DNA damage induced by ROS or genotoxic insults	[145]
8. Tumor-promoting inflammation	E3s	TRAF6/PEL1	TAK1	Lung cancer	Activates TAK1 complex and IKK complex and promotes tumor proliferation	[146]
		BICP0	TRAF6	Cervical Cancer	Promotes K48-ubiquitination of TRAF6 and promotes tumor proliferation	[147]
		TRIM25/TRIM14/MEX3c/RNF135	RIG-I	Breast cancer	Promotes polyubiquitination of RIG-I and promotes tumor proliferation	[148]
		TRIM22	NOD2/NF-κB pathway	Endometrial cancer	Inhibits tumor progression through the NOD2/ NFκB pathway	[149]
		TRAF3	NF-κB	Gastric cancer	Inhibits tumor proliferation	[150]
		LUBAC	NF-κB	Lymphoma	Enhances NF-κB activation	[29]
		LUBAC	NEMO	Breast cancer	Promotes breast cancer development	[30]
		26S proteasome	MDA-7/IL-24	Breast cancer/Lung cancer	Ubiquitinates MDA-7/IL-24 and reduces its antitumor activity	[151]
		OTULIN	M1-linked polyubiquitin signaling	Hepatocellular carcinoma	Promotes tumor growth	[152]
		USP 7	MDM2	Breast cancer	Impairs DDR and promotes cancer progression	[144]
9. Reprogramming energy metabolism	E3s	TRIM36	HK2	Prostate cancer	Inhibits the neuroendocrine differentiation of prostate cancer	[153]
		MARCH8	HK2	Colorectal cancer	Inhibits glycolysis	[154]
		FBW7	C-Myc	Oral squamous cell carcinoma	Inhibits oral squamous cell carcinoma	[155]
		STUB1	PKM2	Colorectal cancer	Inhibits the progress of colorectal cancer	[156]
		TRIM9	PKM2	Triple-negative breast cancer	Promotes glycolysis	[157]
		TRIM29	PKM2	Colorectal cancer	Promotes colorectal cancer carcinogenesis	[158]
		TRIM35	PKM2	Breast cancer	Inhibits the malignant behaviour of breast cancer	[159]
		DLG4	G-6-PD	Colorectal cancer	Inhibits the progress of colorectal cancer	[160]
		STUB1	GDH1	Lung adenocarcinoma	Inhibits the proliferation of cancer cells and cancer growth	[65]
		RNF213	GDH1	Kidney renal clear cell carcinoma	Maintains the survival of cancer cells after amino acid deprivation	[161]
		Trib1-COP1	ACC1	Myeloid leukemia	Promotes the occurrence of myeloid leukemia	[162]
		FBXW7β	FASN	Colorectal cancer	Promotes the growth of colorectal cancer	[163]
		TRIM21	GAC	Non-small cell lung cancer	Promotes the occurrence of non-small cell lung cancer	[164]
	DUBs	USP7	HK2	Gastric cancer	Promotes aerobic glycolysis	[165]
		OTUB1	C-Myc	Breast cancer	Inhibits oral squamous cell carcinoma	[166]
		USP7	C-Abl	Non-small-cell lung cancer	Promotes glycolysis and survival of non-small cell lung cancer cells	[167]
		TRAP1	PFK1	Colorectal cancer	Enhances Warburg metabolism	[168]
		DDX39B	PKM2	Colorectal cancer	Promotes the progress of colorectal cancer	[156]
		OTUB2	PKM2	Colorectal cancer	Exacerbates the progression of colorectal cancer by promoting PKM2 activity and glycolysis	[11]
		PSMD14	PKM2	Ovarian cancer	Promotes ovarian cancer progression by decreasing enzymatic activity of PKM2	[169]
		USP35	PKM2	Hepatocellular carcinoma	Promotes hepatocellular carcinoma progression	[170]
		USP15	GS	Multiple myeloma	Promotes amino acid metabolism	[171]
		USP22	PPARγ	Hepatocellular carcinoma	Promotes tumorigenesis	[172]
10. Avoiding immune destruction	E3s	SPOP protein	PD-L1	Primary human prostate cancer	Promotes degradation of PD-L1	[173]
		SPOP protein	PD-L1	Colorectal cancer	Mediates degradation of PD-L1	[174]
		SPOP protein	PD-L1	Breast cancer	Ubiquitinates PD-L1	[175]
		FBW7	NFAT1	Metastatic renal cell carcinoma	Reduces the expression of PD-L1 by down-regulating NFAT1	[176]
		FBXO38	PD-1	Melanoma	Regulates PD-1 on the cell surface through the polyubiquitination linked with K48	[177]
		FBXO22	PD-L1	Non-small cell lung cancer	Promotes ubiquitination and degradation of PD-L1	[178]
		NEDD4	PD-L1	Urothelial carcinoma	Catalyzes the polyubiquitination of K48 linkage in PD-L1	[179]
		Cbl-b	TCR	Lymphoma	Downregulates TCR expression	[180]
		HERC2	JAK2/STAT3 pathway	Hepatocellular carcinoma	Mediates immune escape through the JAK2/STAT3 pathway	[181]
		RNF31	PD-L1	Triple-negative breast cancer	Inhibits the expression of PD-L1 by inhibiting the Hippo/YAP/PD-L1 axis	[182]
		RNF5	PTEN	Pancreatic ductal adenocarcinoma	Promotes tumor advancement	[183]
		A20	Snail1	Breast cancer	Promotes tumor metastasis	[184]
		TRAF6	CTLA-4	Melanoma	Promotes the ubiquitination and degradation of CTLA-4	[185]
	DUBs	USP7	PD-L1	Gastric cancer	Interacts with PD-L1 to stabilize PD-L1	[186]
		USP22	PD-L1	Liver cancer	Interacts with the C terminus of PD-L1, inducing its deubiquitination and stabilization	[187]
		USP22	PD-L1	Non-small cell lung cancer	Regulates the level of PD-L1 protein through USP22-CSN5 -PD-L1 axis	[188]
		CSN5	PD-L1	Breast cancer	Interacts with PD-L1 and deubiquitinates PD-L1 protein	[189]
		USP8	PD-L1	Pancreatic cancer	Deubiquitinates PD-L1	[190]
		USP14	IDO1	Colonic adenocarcinoma	Stabilizes IDO1 and reduces anti-PD-1 responsiveness	[191]
		USP12	PD-1	Lung cancer	Deubiquitinates PD-1	[192]
		USP9X	PD-L1	Oral squamous cell carcinoma	Combines with PD-L1 to induce its ubiquitination and stabilize its protein expression in oral squamous cell carcinoma	[193]
		USP3	SUZ12	Gastric carcinoma	Stabilizes SUZ12 through deubiquitination to promote EMT of tumor cells	[194]
		USP11	Snail	Breast cancer	Deubiquitinates Snail and enhances tumor EMT and metastatic capacity	[195]
		USP11	TGF-βRII	Breast cancer	Promotes breast cancer metastasis by stabilizing TGF-βRII	[196]
		USP26	SMAD7	Glioblastoma	Negatively regulates TGF-β signaling by deubiquitinating and stabilizing SMAD7	[197]
		USP15	TGF-β I	Glioblastoma	Enhances the tumorigenic effect of TGF-β in glioblastoma	[198]
		USP4	TGF-β I	Breast cancer	Maintains the stability of TGF-βRI	[199]
		USP27X	Snail1	Breast cancer/Pancreatic cancer	Maintains the stability of Snail1	[200]
		DUB3	Snail1	Breast cancer	Inhibits breast cancer invasion and metastasis by promoting Snail1 degradation	[201]
11. Unlock surface plasticity	E3s	MDM2	p53	Dedifferentiated liposarcoma	Targets tumor suppressor p53	[202]
		CYLD	NOX4	Abdominal aortic aneurysm	Ubiquitinates NOX4	[203]
		USP38	FASN	Gastric cancer	Increases the production of triglycerides	[204]
12. Nonmutational epigenetic reprogramming	E2	RAD6a	H2b	Esophageal squamous cell carcinoma	Promotes the proliferation of cancer cells	[205]
	E3s	RNF180	DNMT1	Gastric cancer	Inhibits the proliferation of cancer cells	[206]
		RNF180	DNMT3A	Gastric cancer	Inhibits the vitality and motility of cancer cells	[207]
		DDB1-Cul4A	H2A	Osteosarcoma	Inhibits osteosarcoma progression	[208]
		SCF^FBW7^	Brg1	Gastric cancer	Promotes cancer metastasis	[127]
		Β-TRCP	ARID1A	Gastric cancer	Promotes the destruction of cancer cells	[209]
		SCF	ARID1A	Hepatocellular carcinoma	Enhances the growth of cancer cells in vitro and tumor growth in vivo	[186]
		CHIP	INO80	Colorectal cancer	Achieves effective DNA replication	[210]
	DUBs	USP7	FBP1	Pancreatic cancer	Increases the sensitivity of pancreatic cancer to PARP inhibitors	[211]
		USP22	H2A	Osteosarcoma	Promotes the progress of osteosarcoma	[208]
		OTUD6A	Brg1	Prostate cancer	Promotes tumorigenesis	[212]
13. Polymorphic microbiomes	E3s	PSMB4	NIK/NF-κb pathway	Colorectal cancer	Activates inflammatory response through NIK/NF- κB pathway	[213]
		HECTD2	EHMT2	Colorectal cancer	Promotes proteasomal degradation of EHMT2	[214]
14. Senescent cells	E3	MDM2	p53	Multiple cancers	Under low-stress conditions, p53 will initiate repair and cell cycle arrest mechanisms that will promote cell survival Under acute stress conditions, p53 eliminates the damaged cells from the proliferative pool through apoptosis and senescence	[214]

#### MAPK signaling pathway

The MAPK pathway includes the RAS-RAF-MEK-ERK pathway and the JNK and p38 pathways [215]. In the ERK1/2 signaling pathway, E3 ligases RNF149 and FBXW7 regulate the stability of B-Raf in colon adenocarcinoma (COAD), leading to its degradation. This degradation inhibits ERK1/2 signaling and tumor cell growth [216, 217]. Additionally, the deubiquitinating enzyme USP10 protects C-Raf from degradation in ectopic endometrial stromal cells [218]. The UPS also regulates the ERK signaling pathway by affecting MEK1/2 expression, with USP21 involved in maintaining MEK2 stability and activating ERK signaling in hepatocellular carcinoma. The high expression of USP21 in hepatocellular carcinoma is associated with a lower survival rate among hepatocellular carcinoma patients. The research identified new clinical treatment strategies targeting the USP21-MEK2 interaction and its functions [91]. Activation of the MAPK pathway is known to promote the progression of hepatocellular carcinoma [219]. Targeted therapies against the MAPK pathway have become a focal point, with inhibitors targeting this pathway currently undergoing clinical trials [220, 221]. Deubiquitinating enzymes like USP2A, USP8, and USP15 have been identified as crucial modulators that promote MAPK pathway molecules deubiquitination [222]. Interestingly, USP8 knockdown can overcome gefitinib and erlotinib resistance [223, 224]. However, only a few USP8 inhibitors have been identified. Tian et al. discovered that DC-U4106 effectively binds to USP8 with a KD value of 4.7 μM and significantly suppresses breast cancer tumor growth while exhibiting minimal toxicity in a xenograft model [225].

The JNK1/2/3 and p38 signaling cascades involve multiple MAPKKKs and MAPKKs, including MEKK1/2/3/4, TAK1, and ASK1, which can be activated by various stimuli [226]. USP4 stabilizes the TAK1 protein level in ESCC cells through deubiquitination [82]. USP4/TAK1 plays a critical role in the progression of esophageal squamous cell carcinoma (ESCC) by regulating proliferation, migration, and invasion. Silencing USP4 has been shown to inhibit tumor proliferation in ESCC nude mouse models. Moreover, the USP4 inhibitor, Neutral Red, can suppress ESCC progression both in vitro and in vivo [82]. Another UDB molecule, USP15, can also target TAK1 and inhibit the proteasomal degradation of TAK1-binding protein 2 (TA B2) [227]. Apoptosis signal-regulating kinase 1 (ASK1) is a MAPKKK that initiates cell death and inflammatory responses by activating the p38 and JNK signaling pathways [228, 229]. The E3 ligase inhibitor of apoptosis protein (IAP) can directly bind to ASK1 and induce its degradation via the E2 enzyme UbcH5. IAP depletion increases TNF receptor 2 (TNFR2)-mediated activation of p38 and JNK, increasing tumor cell proliferation [230]. These research findings reveal the role of the UPS in regulating important signaling pathways in various cancers, providing new insights for the future development of cancer therapeutics.

#### PI3K/AKT/mTOR signaling pathway

AKT, also known as phosphokinase B (PKB), plays a central role in the PI3K/AKT/mTOR signaling pathway [231]. Ubiquitin-conjugating enzyme E2S (UBE2S), mitochondrial ubiquitin ligase activator NF-κB (MULAN), ret finger protein 2 (RFP2), breast cancer susceptibility gene 1 (BRCA1), speckle-type POZ protein (SPOP), TNF receptor-associated factor 4 (TRAF4), and F-box and leucine-rich repeat protein 18 (FBXL18), regulate AKT through ubiquitination, affecting its degradation or activation in various cancers (Table 1) [74–76, 78, 79, 232]. UBE2S has been shown to be associated with AKT phosphorylation [233]. One study found that UBE2S is highly expressed in epithelial ovarian cancer and induces cisplatin resistance by activating the PI3K/AKT/mTOR signaling pathway and inhibiting autophagy. Knocking down of UBE2S can inhibit the proliferation and migration of cisplatin-resistant ovarian cancer cells, providing new insights for the evaluation and treatment of high-risk ovarian cancer patients with cisplatin resistance [234]. SPOP, an E3 ligase, inhibits the activity of AKT kinase and its oncogenic function by mediating the ubiquitination and degradation of phosphatidylinositol-dependent protein kinase 1 (PDK1) (upstream protein of AKT). Cancer patients with PDK1 mutations exhibit oncogenic effects by evading SPOP recognition. This could be an attractive therapeutic direction [235].

The mTOR signaling pathway plays a vital role in regulating essential cellular functions such as cell growth, autophagy, metabolism, and DNA damage [236]. The lipid phosphatase PTEN can antagonize PI3K [237]. In cervical cancer cells, the E3 ligase makorin ring finger protein 1 (MKRN1) ubiquitinates and degrades the PTEN protein. In cervical cancer patients exhibiting high expression levels of MKRN1, the protein level of PTEN is found to be lower, which is associated with a decreased 5-year survival rate [69]. Additionally, another study identified that the deubiquitinase OTUD3 interacts with the substrate KPTN to regulate the mTORC1 signaling pathway, significantly inhibiting tumor cell proliferation and growth. By uncovering OTUD3’s essential role in cancer, this research provides crucial insights for developing novel cancer treatment strategies targeting OTUD3 or its regulatory pathways [238]. Additionally, the E3 ligase FBX8 partially achieves its tumor suppressor function by degrading mTOR in colorectal cancer. Low expression levels of FBX8 are correlated with poor prognosis in colorectal cancer patients [71]. In prostate adenocarcinoma (PRAD), the E3 ligase sensitive to apoptosis gene (SAG) targets DEPTOR for degradation, activating the mTORC2/AKT signaling pathway and promoting tumorigenesis. The SAG conditional KO mouse model was employed with PTEN deletion in the prostate to assess the in vivo function of SAG in prostate cancer development, indicating that targeting the SAG E3 ligase could be beneficial in prostate cancer therapy [72]. These experiments demonstrate that targeting the E3 ligases that regulate key proteins in the PI3K/AKT/mTOR signaling pathway offers promising therapeutic avenues for various cancers, providing a new direction for developing more effective cancer treatment strategies.

### Evading growth suppressors

Inactivation of tumor suppressors eliminates the negative regulation of cell growth and proliferation to promote cancer development [239]. In addition to inducing and maintaining positive growth-stimulating factors, cancer cells must evade growth suppressors. Typical tumor suppressors encode retinoblastoma (RB) and p53 proteins, which regulate cell proliferation and apoptosis (Table 1) [6, 103].

#### p53

p53 has the highest frequency of mutations in human cancers and is usually expressed at low levels in cancer cells [172]. p53 regulates the cell cycle, induces apoptosis in response to DNA damage, and contributes to genomic stability by promoting DNA repair [172]. MDM2 functions as a p53 monoubiquitinating E3 ligase, facilitating the ubiquitination and subsequent degradation of p53 [104]. Currently, activating p53 by antagonizing MDM2 involves several approaches: (a) reducing MDM2 expression; (b) inhibiting its ubiquitin ligase function; and (c) blocking interactions between MDM2 and p53 [104]. The strategy of disrupting MDM2-p53 interactions using small molecules has been extensively pursued. For instance, AMG232 triggered apoptosis and inhibited cell proliferation in glioblastoma and multiple myeloma. The observations also indicated a great specificity for p53 wild-type cells compared to p53 mutant stem cells in glioblastoma [240]. AMG232 has also been studied in clinical trials [241]. E3 ligases TRIM28 and TRIM31 are also reported to promote p53 degradation. In osteosarcoma cells, TRIM28 cooperates with MDM2 to regulate the ubiquitination and degradation of p53, promoting tumor proliferation [95]. A high level of TRIM31 correlates with shorter overall survival (OS) in lung cancer patients [242]. Elevated levels of TRIM31 are associated with more aggressive characteristics and unfavorable outcomes in pancreatic cancer patients. Inhibition of TRIM31 increases the sensitivity of gemcitabine in pancreatic cancer cells, indicating suppressing TRIM31 could be an effective approach to improve the efficacy of gemcitabine in overcoming chemotherapy resistance in pancreatic cancer [243]. Furthermore, USP7 is identified to directly deubiquitinate p53, inhibiting tumor proliferation. High levels of USP7 and MDM2 are implicated in the onset and development of various cancers, playing a critical role by suppressing p53 activities. Inhibiting these proteins can reactivate p53 pathways, leading to the halting of the cell cycle and programmed cell death. Studies emphasize the pharmacological properties, potential therapeutic uses, and the action mechanisms of small molecule inhibitors targeting USP7 and MDM2 [104]. Moreover, USP25 has been shown to be an important upstream regulator of the MDM2-p53 signaling pathway and has the potential to be a novel target gene for developing new therapeutic applications [244].

#### RB

The dysregulation of the RB pathway is frequently observed in cancer. The impairment of RB function, frequently due to mutations or mechanisms that induce hyperphosphorylation, allows uncontrolled cell cycle progression [245]. This process can result in excessive cell proliferation and contribute to tumor development. In addition to being phosphorylated, RB can be ubiquitinated, sumoylated, acetylated, or methylated [246]. TRIM28 binds to the phosphorylated RB protein (p-RB), promoting its ubiquitination and degradation [95]. On the other hand, SETDB1, a binding partner of TRIM28, protects p-RB from degradation, which is particularly notable in prostate cancer [247]. Inhibiting SETDB1 expression reduces tumor growth but accelerates the degradation of RB protein. Notably, combined use with the CDK4/6 inhibitor palbociclib can block SETDB1 inhibition-induced RB degradation and demonstrate stronger anticancer effects. These research findings reveal the potential value of using a combination strategy of CDK4/6 and SETDB1 inhibition to reduce RB degradation and suppress cancer growth [248].

#### ADP-ribosylation Factor (ARF)

ARF is a tumor suppressor encoded by the cyclin-dependent kinase inhibitor 2A (CDKN2A) locus and primarily exerts its tumor suppressive effects through the MDM2-P53 axis [98]. Under normal conditions, oncogenic signals induced by MYC, RAS, and E2Fs lead to the upregulation of ARF. ARF subsequently inhibits MDM2, thereby activating the tumor suppressor function of p53 [98, 249]. ARF function, stability, and cellular localization are tightly regulated by posttranslational modifications such as phosphorylation and ubiquitination [98]. Elongin B (ELOB), as a core component of the Cullin2-RBX1-ELOB E3 ligase (CRL2) complex, regulates ubiquitination and degradation of the oncoprotein p14/ARF [250]. Research indicates that a peptide strongly adheres to the ELOB/C dimer, disrupting the binding of ELOB/C to its binding molecules. Treatment of cancer cells with this peptide inhibitor led to reduced cell survival, heightened apoptosis, and altered gene activity. Consequently, these findings suggest that targeting the BC-box-binding pocket of ELOB/C is a viable method for disrupting its activity and inhibiting the proliferation of cancer cells [251]. Prame is overexpressed in tumor tissues compared to paired adjacent tissues and is associated with poor prognosis in cancer patients. As a substrate recognition receptor protein of Cullin RING E3 ligases (CRLs), Prame regulates the ubiquitination and subsequent degradation of ARF through the Cullin2-RBX1-ELOB E3 ligase complex, making it a potential novel therapeutic target [252].

### Resist cell death

**Fig. 4:**
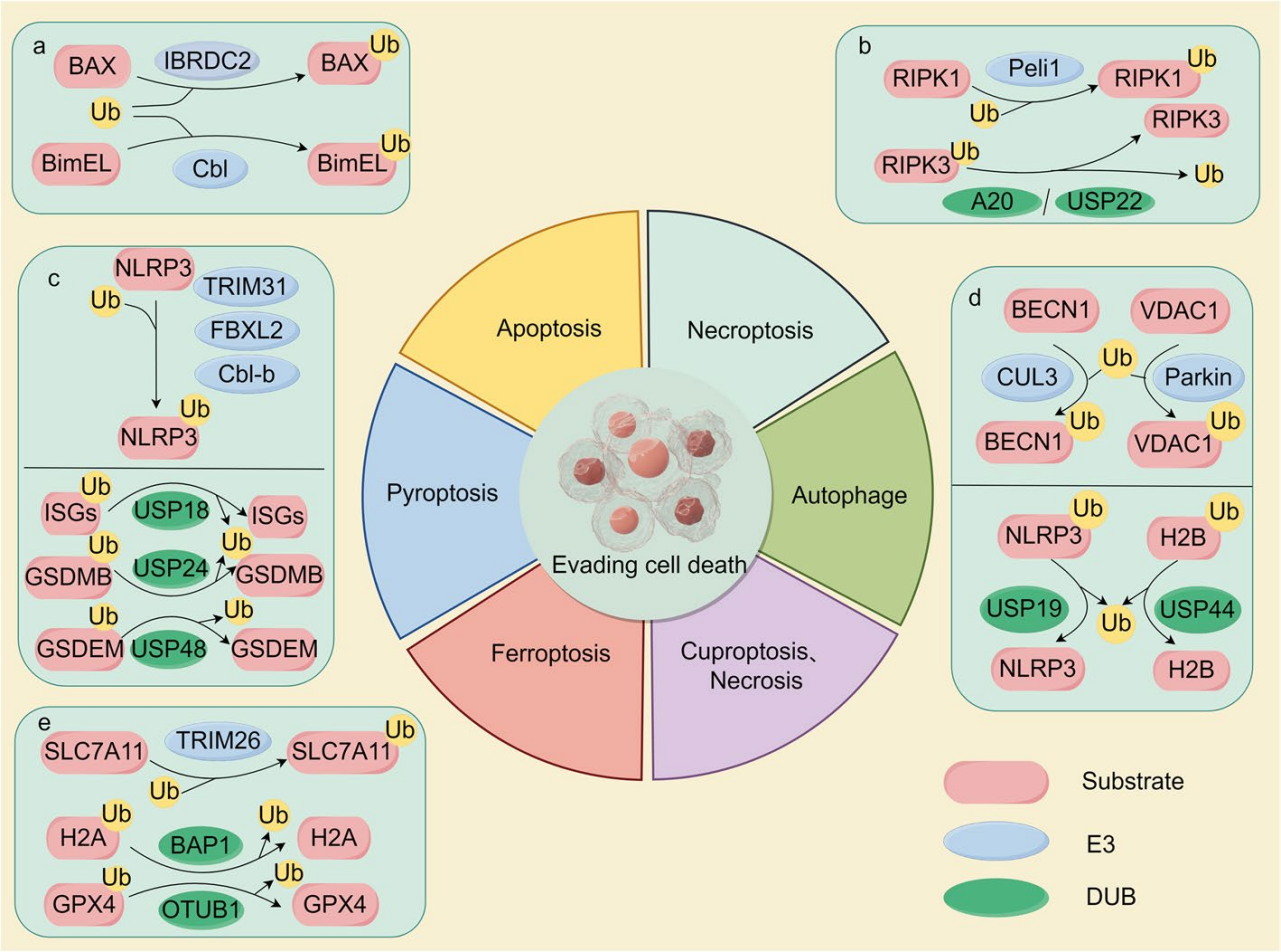
The ubiquitin–proteasome system (UPS) plays a vital role in resisting cell death through various mechanisms. **a** IBR domain containing 2 (IBRDC2) can target Bcl-2-associated X protein (BAX) for ubiquitination and degradation, which can prevent mitochondrial outer membrane permeabilization (MOMP) induced by active BAX and reduce cell apoptosis. Additionally, Cbl can target BimEL for ubiquitination and degradation, thereby inhibiting apoptosis. **b** A20 and USP22 can deubiquitinate receptor-interacting protein kinase 3 (RIPK3) to stabilize it, thus suppressing TNF-α-induced necroptosis. Pellino1 (Peli1) can mediate K63 ubiquitination on K115 of RIPK1 in a kinase-dependent manner, promoting the formation of necrosomes and facilitating necroptosis. **c** Tripartite motif 31 (TRIM31), F-box and leucine-rich repeat protein 2 (FBXL2), and casitas b-lineage lymphoma-b (Cbl-b) promote NLRP3 inflammasome protein 3 (NLRP3) polyubiquitination at different sites, thereby inhibiting the process of pyroptosis. USP18 inhibits pyroptosis in cancer cells via enhancing interferon-stimulated genes (ISGs), while USP48 promotes pyroptosis by stabilizing gasdermin E (GSDEM), and USP24 promotes pyroptosis by stabilizing gasdermin B (GSDEB). (d) Cullin3 (CUL3) and Parkin are responsible for ubiquitinating beclin 1 (BECN1) and voltage-dependent anion channel 1 (VDAC1), respectively, whereas USP19 and USP44 can deubiquitinate NLRP3 and H2B, respectively. **e** TRIM26 targets solute carrier family 7 member 11 (SLC7A11) for ubiquitination and degradation, promoting cellular ferroptosis. BRCA1-associated protein 1 (BAP1) removes H2A ubiquitination from the SLC7A11 promoter, resulting in decreased cystine uptake and increased ferroptosis. OTU deubiquitinase ubiquitin aldehyde-binding 1 (OTUB1) promotes glutathione peroxidase 4 (GPX4) deubiquitination, inhibiting ferroptosis in gastric cancer cells

Cell death is a normal physiological process in all living organisms and plays essential roles in embryonic development, homeostatic maintenance, aging, and immune coordination [253]. Cell death includes apoptosis, necrotizing apoptosis, pyroptosis, autophagy, ferroptosis, cuproptosis [182, 253, 254]. Ubiquitination also plays an essential role in resisting cell death. In the following paragraphs, we will briefly introduce the types of cell death and describe the functions of ubiquitination and deubiquitination in these types of cell death (Fig. 4).

#### Apoptosis

Apoptosis is the most common noninflammatory form of programmed cell death [182, 255]. It produces remnants of apoptotic cells, such as cytoplasm, organelles, and the contents of some nuclei, which are randomly sorted into each apoptotic body [105]. Two main pathways of apoptosis have been identified: the intrinsic apoptosis pathway and the extrinsic pathway initiated by death receptors [105, 182]. The intrinsic and extrinsic apoptosis pathways depend on the activation of the caspase family [256]. BAX is the main pro-apoptotic executioner protein. The E3 ligase IBR domain containing 2 (IBRDC2) can target BAX for ubiquitination-mediated degradation, thereby preventing mitochondrial outer membrane permeabilization (MOMP) induced by active BAX [105]. When inducing apoptosis, IBRDC2 accumulates in BAX-rich mitochondrial structures, allowing the accumulation of BAX to occur simultaneously with its activation [106]. BimEL, belonging to the Bcl-2 protein family, crucially promotes apoptosis by inducing mitochondrial outer membrane permeabilization (MOMP) and activating the caspase cascade. The E3 ligase Cbl can degrade the extralong splice variant of Bim (BimEL) and is cell type-specific [105]. Icotinib is a specific tyrosine kinase inhibitor (TKI) targeting the epidermal growth factor receptor (EGFR). Treatment with Icotinib significantly reduces the levels of p-EGFR (phosphorylated EGFR), p-ERK (phosphorylated extracellular signal-regulated kinase), and c-Cbl in HCC827 lung cancer cells, leading to inhibited proliferation and induced apoptosis of HCC827 lung cancer cells [257]. Aurora A phosphorylates BimEL, enhancing binding to the F-box protein β-transducin, which contains the E3 ligase. This interaction facilitates the ubiquitination and subsequent degradation of BimEL [258]. The E3 ligases mentioned above all decrease the levels of pro-apoptotic proteins; therefore, finding drugs that can inhibit the binding affinity of these ligases with their target proteins can maintain a certain level of apoptosis in cancer cells, effectively inhibiting the development of cancer.

#### Necroptosis

Necroptosis, a type of programmed cell death, involves cell and organelle swelling, membrane rupture, and the release of cellular contents [259]. Necrotizing apoptosis is a receptor-interacting protein kinase 1 (RIPK1)-RIPK3-mixed lineage kinase domain-like protein (MLKL) pathway triggered by death and Toll-like receptor 3/4 [260]. Pellino1 (Peli1) can mediate K63 ubiquitination on K115 of RIPK1 in a kinase-dependent manner, promoting the formation of necrosomes and facilitating necroptosis [23]. OTULIN can remove the M1 chain from the necroptosis pathway, enhancing TNF-α-induced necrotizing apoptosis [25]. Ubiquitin-editing enzyme A20 removes K63-linked ubiquitin chains from RIPK3 [22], inhibiting RIPK3 ubiquitination and reducing RIPK1:RIPK3 interactions. This inhibition effectively restrains TNF-α-induced necrotizing apoptosis, which can be reversed by USP22 [18]. Interestingly, several cases of solid tumors with high A20 expression are associated with lower survival rates [261]. Knocking down of A20 reduces cell growth and enhances sensitivity to agents that induce apoptosis [262]. Moreover, researchers found that A20 plays a vital role in drug resistance, and they established a direct link between elevated A20 levels and increased in vitro resistance to tumor necrosis factor-related apoptosis-inducing ligand (TRAIL) [263].

#### Pyroptosis

Pyroptosis is the programmed death of cells caused by the activation of inflammasome sensors. It results in plasma membrane lysis, cell swelling, chromatin fragmentation, and the release of intracellular proinflammatory contents [253, 264]. Pyroptosis mainly relies on the activation of caspase family proteins by inflammasomes to cause various physiological responses, and NOD-like receptor protein 3 (NLRP3) is a typical inflammasome [265]. The E3 ligase TRIM31 can act as a feedback inhibitor for the NLRP3 inflammasome, directly binding to NLRP3, promoting K48-linked polyubiquitination, and facilitating its degradation via the proteasome [96]. E3 ligase F-box and leucine rich repeat protein 2 (FBXL2) interact with Trp73 within the NLRP3 protein specifically by targeting the ubiquitination and subsequent degradation of Lys689 [266]. The E3 ligase Cbl-b binds to K63 ubiquitin chains on the leucine-rich repeat domain (LRR) of NLRP3 and then targets the K496 site to link with K48 ubiquitin chains and mediate proteasomal degradation [267, 268]. Notably, by comparing the effects of the NLRP3 inducer Nigericin across various tumor types and normal fibroblast controls, it was discovered that Nigericin may represent a novel therapeutic approach for controlling the growth of tumors that produce low levels of IL-1β and IL-18 [269]. According to recent literature, several DUBs were reported to regulate pyroptosis in cancer, including USP18 [270], USP24 [112], and USP48 [113]. Mechanistically, USP18 inhibits pyroptosis in cancer cells via enhancing ISGs, while USP48 promotes pyroptosis by stabilizing gasdermin E (GSDEM). An in vivo study indicates that upregulating USP48 can enhance the antitumor activity of PD-1 inhibitor [113], suggesting that USP48 activation pharmacologically could be a promising approach to enhance cancer cell sensitivity to pyroptosis and improve immunotherapy outcomes.

#### Autophagy

Autophagy is initiated in response to various signals, including nutrient deprivation, the absence of growth factors, hypoxia (low oxygen levels), and exposure to pathogens [271]. Autophagy has been shown to play a critical role in tumor maintenance, even with elevated basal autophagy levels in many tumors under nutrient-adequate conditions. Autophagy inhibition or systemic autophagy inhibition in tumor cells disrupts tumor metabolism, resulting in antitumor effects [272]. Autophagy can promote tumor cell survival by providing nutrients during periods of stress, such as nutrient deprivation or hypoxia. USP19 plays a significant role in autophagy regulation. It cleaves the ubiquitin chain of NLRP3, inhibiting proteasomal degradation and transforming NLRP3 from a proinflammatory to an anti-inflammatory state [273]. Beclin 1 (BECN1) is an important member of the autophagy-related protein family, primarily involved in regulating the formation of autophagic vesicles [109]. In various cancers such as breast cancer, ovarian cancer, and colorectal cancer, the expression level of BECN1 is significantly reduced, which is positively correlated with poor prognosis for patients [109]. In breast cancer and ovarian cancer, the E3 ligase cullin-3 (CUL3) interacts with BECN1, promoting its K48 ubiquitination and downregulating BECN1, ultimately enhancing tumor cell proliferation and resulting in a poor prognosis [274]. Therefore, searching for inhibitors of CUL3 may be an important approach for treating breast cancer and ovarian cancer. A research team has also targeted the functions of Kelch-like (KLHL) family proteins, which are substrate adaptor proteins of Cullin3-RING ligase (CRL3), in order to disrupt the function of CRL3 [97].

#### Ferroptosis

Ferroptosis is a recently discovered form of cellular death triggered by the excessive accumulation of iron-dependent lipid peroxidation products [275, 276]. It primarily occurs through exogenous and endogenous pathways involving transporter-dependent and enzyme-regulated mechanisms [276]. The regulation of ferroptosis involves a balance between tumor promotion and inhibition. Genetic ablation of solute carrier family seven member 11 (SLC7A11) or glutathione peroxidase 4 (GPX4) induces ferroptosis in cancer cells, leading to significant tumor suppression [277]. The E3 ligase TRIM26 mediates the ubiquitination of SLC7A11, targeting it for proteasomal degradation and inducing ferroptosis. This process inhibits hepatic stellate cell activation and reduces liver fibrosis [110]. Searching for agonists of TRIM26 might be a strategy for treating early-stage liver cancer. The deubiquitinase BRCA1-associated protein 1 (BAP1) plays an essential role in regulating ferroptosis by removing H2A ubiquitination (H2Aub). BAP1 forms a polycomb repressive deubiquitinase (PR-DUB) complex that deubiquitinates H2Aub on the SLC7A11 promoter, leading to decreased cystine uptake and increased ferroptosis [278]. DUB enzyme OTU deubiquitinase ubiquitin aldehyde-binding 1 (OTUB1) promotes GPX4 deubiquitination, thereby inhibiting ferroptosis in gastric cancer cells [279]. OTUB1 is a distinctive target because of its conventional and unconventional functions. The compound PR-619 is anticipated to decrease OTUB1 activity, as evidenced by its capacity to prevent OTUB1 from binding to an active site probe [114].

### Enabling replicative immortality

Telomeres, conserved nucleoprotein structures found at the termini of linear eukaryotic chromosomes, consist of repetitive sequences (TTAGGG)n in humans [280]. They interact with six protein species that form a “shelterin complex” [281]. As a result of repeated rounds of replication in eukaryotic cells, telomeres are shortened continuously. Therefore, during tumor development, cells must utilize a telomere DNA maintenance mechanism (TMM) to counteract telomere shortening, protect telomeres from the influence of the DNA damage repair system, and avoid telomere-mediated aging and apoptosis [282]. Changes in telomere structure are intricately linked to the onset and progression of tumors. Telomere repeats, and the involvement of TRF1 and TRF2 serve as guardians of telomeres, whose expression levels are disrupted across diverse cancer forms [283]. In renal cell carcinoma (RCC), the telomere proteins TRF1 and TRF2 are overexpressed, and their inhibition by siRNAs can induce apoptosis, reducing cell proliferation and migration [284].

Recent studies have shown that the ubiquitin mechanism can regulate elements of telomeres. Furthermore, the UPS influences cancer progression by impacting telomeres. Ubiquitin-mediated degradation of telomere associated protein TRF1 levels is facilitated by E3 ligases such as repeatability limit (RLIM), β-TRCP1, and FBX4 (Table 1) [115–117]. Clinical studies have shown that TRF1 upregulation in glioblastoma multiforme (GBM) contributes to tumor initiation and progression. This was demonstrated by the inhibition of tumor growth and extended survival in GBM mouse models following brain-specific TRF1 genetic deletion. Additionally, chemical inhibitors of TRF1 in human GBM cells blocked tumor sphere formation and slowed growth in patient-derived GSC xenografts [285]. These studies suggest the direction of future clinical research on ubiquitination. Conversely, members of the chromatin-modifying complex family, such as general control nonderepressible-5 (GCN5) and USP22, have been reported to facilitate the deubiquitination of TRF1 [286]. GCN5 is necessary for the binding of USP22 to Spt-Ada-Gcn5 acetyltransferase (SAGA) complexes, enabling the deubiquitination of TRF1 and preventing its turnover [286]. Research has demonstrated that eliminating USP22 from pancreatic tumor cells enhances the immune response by decreasing suppressive myeloid cells and increasing cytotoxic T cells and natural killer cells. Additionally, USP22 influences the cancer cell transcriptome, thereby modifying the immune tumor microenvironment. Targeting USP22 in pancreatic cancer can enhance the effectiveness of immunotherapy and improve treatment outcomes [287].

TPP1, another shelterin protein subunit, also undergoes ubiquitin-mediated proteolysis, which has been evidenced by the stability of TPP1 protein levels after proteasome inhibition. In mice, the stabilization of TPP1 at telomeres requires its ubiquitination by the E3 ligase RNF8 [120]. Recent research has indicated that F-box and WD repeat domain-containing 7 (FBW7) can promote cell senescence and tissue fibrosis by facilitating telomere decapitation [118]. The deubiquitinase USP7 interacts with human TPP1 and removes ubiquitin chains. Although the degradation of USP7 does not impact the level of TPP1 regulated by the proteasome, USP7 might interact with other deubiquitinases redundantly to stabilize TPP1 [288]. By now, P22077 has been extensively studied and has become a prevalent tool compound for inhibiting USP7 in biological research. For example, it has been shown that P22077 can effectively trigger p53-dependent apoptosis in neuroblastoma (NB) cells and markedly reduce tumor growth in xenograft models of three NB cell types [289]. Additionally, certain natural compounds have also been identified as USP7 inhibitors. Notably, Spongiacidin C, a pyrrole alkaloid from the marine sponge Stylissa massa, was identified as a USP7 inhibitor with an IC50 of 3.8 μM, though its precise in vivo effects need further investigation [290].

### Inducing angiogenesis

Tumor angiogenesis refers to the process of forming new blood vessels within and surrounding tumors. This phenomenon is critical for tumor growth and progression, as it provides the necessary nutrients and oxygen supply to sustain rapidly dividing cancer cells [291]. The rapid growth of tumors leads to areas with low oxygen concentrations, known as hypoxia. In response to hypoxia, cells release hypoxia-inducible factors (HIFs) that stimulate the expression of proangiogenic factors, including vascular endothelial growth factor (VEGF), to form new blood vessels [292]. The specific prolyl-4-hydroxylase enzyme continuously hydroxylates HIF1α. Once hydroxylated, HIF1α is recognized by the E3 ligase complex, leading to its polyubiquitination and subsequent degradation by the proteasome [291, 293]. VEGF is highly expressed in most human tumors [294]. Under normoxic conditions, the E3 ligase von hippel lindau (VHL) ubiquitinates HIF-1, thereby preventing the dimerization and binding of HIF-1 to the promoter of the VEGF gene and inhibiting its transcription and translation. Under hypoxic conditions, HIF-1 dimerizes and stimulates VEGF production and angiogenesis [295]. By regulating the levels of HIF1α, this process directly influences the cellular response to low oxygen conditions. A study has revealed that USP22 promotes the stemness of hepatocellular carcinoma induced by hypoxia-inducible factors through a HIFα/USP22 positive feedback loop after TP53 inactivation [296]. This process contributes to promoting angiogenesis, tumor invasion and metastasis, and tumor drug resistance [296]. The research team achieved high tumor suppression and increased sensitivity to sorafenib in mice with hepatocellular carcinoma by targeting USP22 with a lipid-polymeric complex. This further indicates that USP22 is a highly promising therapeutic target for hepatocellular carcinoma [296]. Additionally, seven in absentia homology 2 (SIAH2) can target HIF for ubiquitination and degradation, thereby modulating the cellular response to hypoxic conditions. HIF-mediated inhibition of nuclear respiratory factor 1 (NRF-1) reduces the transcription of mitochondrial genes and inhibits the activity of the E3 ligase SIAH2 [121].

### Activating invasion and metastasis

The invasion and metastasis of tumors include the movement of tumor cells, infiltration into neighboring tissues, circulation, and extravasation to distant organs, which are the leading causes of cancer-mediated damage to the body [297]. Ubiquitination and deubiquitination are pivotal in numerous protein modification and regulatory processes and often influence tumor invasion and metastasis. This section aims to explore the role of the ubiquitin mechanism in tumor invasion and metastasis and analyze recent findings related to E3 ligases and DUBs and their potential mechanisms (Fig. 3).

#### The role of E3 ligases in cancer metastasis

E3 ligases F-Box and WD repeat domain containing 2 (FBXW2) [122], FBW7 [127], Ub-protein ligase E3C (UBE3C) [131], and F-Box protein 22 (FBXO22) [298] play vital roles in cancer metastasis. For instance, FBXW2 functions as a tumor suppressor by facilitating the ubiquitination and degradation of oncogenic proteins such as SKP2 [123] and β-catenin [122], thus impeding cancer migration, invasion, and metastasis. FBXW2 can be ubiquitinated and degraded as a substrate of β-TrCP1 [123]. Additionally, overexpression of FBXW2 decreases β-catenin-driven transactivation and suppresses invasion, while depletion enhances β-catenin stability and promotes lung cancer metastasis [122]. FBW7 acts as a tumor suppressor by promoting the degradation of cancer-related proteins like Snail [126], Brahma-related gene 1 (Brg1) [127], and YTH N6-methyladenosine RNA Binding Protein F2 (YTHDF2) [128], thereby inhibiting metastasis in various cancers including non-small cell lung cancer, gastric cancer, and ovarian cancer. It also modulates the HIF-1α/CEACAM5 axis in colorectal cancer and potentially predicts immunotherapy response in thymic cancer [129]. In clinical studies, it has been shown that low expression of FBW7 in breast cancer cells leads to resistance to the BET inhibitor JQ1, but combining JQ1 with a Mcl-1 inhibitor can overcome this resistance. This finding suggests that enhancing the effectiveness of BET inhibitors in patients with low FBW7 expression is a promising clinical strategy [299].

UBE3C is a tumor promoter that ubiquitinates substrates such as neuroblast differentiation-associated protein (AHNAK), disrupting the p53-AHNAK complex and enhancing stem cell-like properties in non-small cell lung cancer [300, 301]. It also promotes RCC growth and metastasis by upregulating β-catenin and activating the Wnt/β-catenin pathway [132]. In non-small cell lung cancer, FBXO22 promotes Lys63-linked polyubiquitination of liver kinase B1 (LKB1), reducing its activity and impeding the LKB1-AMPK-mTOR pathway, thereby enhancing cell proliferation. Clinically, elevated FBXO22 levels in lung adenocarcinoma patients indicate a poor prognosis [302]. FBXO22 promotes angiogenesis and tumor cell migration by increasing the levels of vascular endothelial growth factor A and HIF-1α expression [303]. Recent research suggests that FBXO22 may facilitate the ubiquitin-mediated degradation of cyclin G-associated kinase (GAK), thereby inhibiting the proliferation and metastasis of cervical cancer cells [304]. Additionally, clinical studies have shown that FBXO22 negativity significantly affects survival in breast cancer patients, especially those with invasive lobular carcinoma (ILC), and leads to poorer outcomes in patients treated with selective estrogen receptor modulators (SERMs) [305]. These findings suggest the need for tailored therapeutic strategies based on histopathological types when considering adjuvant endocrine therapy.

#### The role of DUBs in cancer metastasis

The deubiquitinating enzymes BRCA1-associated Protein 1 (BAP1), USP7, and USP43 are the primary focus of the discussion below. BAP1, characterized by its UCH domain, is a crucial tumor suppressor across various malignancies. In breast cancer, BAP1 promotes tumorigenesis by stabilizing Kruppel-like factor 5 (KLF5) through deubiquitination, facilitating cell cycle progression, while its depletion inhibits tumorigenesis and lung metastasis [306]. BAP1 holds significant potential in clinical research. A study on Pembrolizumab efficacy in thymic cancer found that PD-L1 expression, along with alterations in genes or pathways like BAP1, may predict patient response or resistance to immunotherapy [307]. USP43 mediates Cav2.2 function by regulating cortical actin stability, extracellular matrix degradation, and migration, with Cav2.2 enhancing USP43 expression through NFAT2 activation, thus promoting breast cancer metastasis [308]. USP43 is markedly expressed in epithelial ovarian cancer, fostering cell proliferation, migration, invasion, and cisplatin resistance by stabilizing HDAC2 and activating the Wnt/β-catenin pathway. These discoveries underscore the clinical importance of USP43 in epithelial ovarian cancer, accentuating its potential as a therapeutic target to manage cancer progression, increase sensitivity to cisplatin chemotherapy, and ultimately enhance patient outcomes [309].

### Genome instability and mutation

Genome instability is the core of carcinogenesis in multicellular organisms and is characterized by a high frequency of mutations in cell lineage genomes. High-frequency DNA damage and epigenetic or mutation-induced reductions of DNA repair gene expression may contribute to genome instability [310, 311].

DDR pathways are complex and intricate. Thousands of endogenous and exogenous DNA damage events occur daily [312, 313]. The MRE11-RAD50-NBS1 (MRN) complex first recognizes the repair factors recruited at DNA fragmentation sites [314]. The E3 ligase RNF126 ubiquitinates meiotic recombination 11 (MRE11) at K339 and K480, activating the DDR and conferring resistance to radiotherapy in triple-negative breast cancer (Table 1) [142]. A member of the PI3/PI4-kinase family, ataxia-telangiectasia mutated (ATM) is a protein kinase that is essential for the cellular response to DNA damage, specifically double-strand breaks (DSBs), and is mainly involved in preserving genomic integrity. [315]. Research has found that the E3 ubiquitin ligase Peli1 is activated by ATM-mediated phosphorylation, promoting the ubiquitination of NBS1 and enhancing the accumulation of ATM and the MRN complex at DSB sites [316, 317]. SAM and HD domain containing protein 1 (SAMHD1) combines with the DSB repair initiator CtBP-interacting protein (CtIP) to promote DNA repair [318]. It is worth noting that USP7 interacts with SAMHD1 and deubiquitinates the K421 site, thus reducing its degradation by the proteasome to stabilize SAMHD1. Consequently, it repairs DNA damage induced by ROS or genotoxic insults, overcoming carcinogenic stress and influencing chemotherapy sensitivity [145].

### Tumor-promoting inflammation

Chronic inflammation is an essential factor in cancer development and is associated with approximately 20% of human cancers [319]. Cancer often occurs in inflamed tissues, suggesting that local inflammation plays an essential role in cancer initiation and progression. Moreover, ubiquitination can contribute to tumors initiated by chronic inflammation through the regulation of transcription factors and cytokines, thereby inducing cancer development, maintenance, and metastasis (Fig. 3) [320].

Chronic inflammation and NF-κB activation are closely associated with cancer progression and spread. Linear ubiquitination of key NF-κB regulators by LUBAC plays an essential role. Abnormally regulated linear ubiquitin signaling is associated with cancer initiation and progression [321]. For example, elevated LUBAC expression enhances NF-κB activation, accelerating the development of somatic mutations and lymphoma pathogenesis [29]. The natural compound thiolutin, which specifically inhibits LUBAC, has been shown to inhibit tumor growth in mouse xenograft models, indicating that LUBAC could be a viable therapeutic target for B-cell lymphoma [29]. However, the deubiquitinase OTULIN negatively regulates linear ubiquitin signaling. In hepatocytes, OTULIN deficiency contributes to hepatocellular carcinoma development [152].

In addition, the upstream signaling activator pattern recognition receptor (PRR) is also regulated by E3 ligases [322, 323]. Toll-like receptors (TLRs) are essential components of the immune system, that can activate NF-κB and induce interferon (IFN) production [323, 324]. Alternatively, the c-Cbl ubiquitin ligase is involved in TRAF6 ubiquitination and negatively regulates NF-κB activity [325]. In the context of nucleotide-binding oligomerization domain (NOD)-like receptor (NLR), overexpression of TRIM22 reduces the occurrence and development of endometrial cancer, and its inhibition is mediated by the NOD-NF-κB pathway, which may be one of the mechanisms of NLR [149], indicating TRIM22 may emerge as a valuable prognostic indicator in endometrial cancer patients. TRIM22 can interact with IKKγ, an upstream molecule in the NF-κB pathway, increasing the K63-linked polyubiquitination of IKKγ, thereby activating the NF-κB pathway in GBM. This study indicates that inhibiting the E3 ligase activity of TRIM22 or blocking its interaction with the IκBα or IKKγ proteins could have significant implications for the development of potential therapeutic drugs for GBM [326]. In addition, NOD1 protects intestinal cells from precancerous lesions by inhibiting the NF-κB signaling pathway through the induction of TRAF3 [150]. Cytokines play a crucial role in the tumor microenvironment, promoting communication between malignant cells and surrounding cells. The UPS can influence cancer progression by regulating cytokines [320]. For example, SAG plays an important role in chronic inflammation-induced cancers by ubiquitylating key apoptotic factors such as SARM and Noxa, regulating the ratio of pro- and antiapoptotic factors. Therefore, SAG-UPS may serve as an early diagnostic marker for liver cancer and a potential target for therapeutic development [327, 328]. In conclusion, chronic inflammation, closely linked to aberrant ubiquitination pathways, is a significant driving force in cancer initiation and progression, highlighting potential targets for future cancer treatments and prognostic indicators.

### Reprogramming energy metabolism

#### Glucose metabolism

Cancer tissues need to be reprogrammed in terms of both matter and energy to maintain or further enhance the progress of cancer. Otto Warburg was the first to discover the unique metabolism of cancer cells. He observed that cancer cells exhibit a greater tendency toward glycolysis under aerobic conditions, which is called the “Warburg effect” or “aerobic glycolysis” [329].

**Fig. 5:**
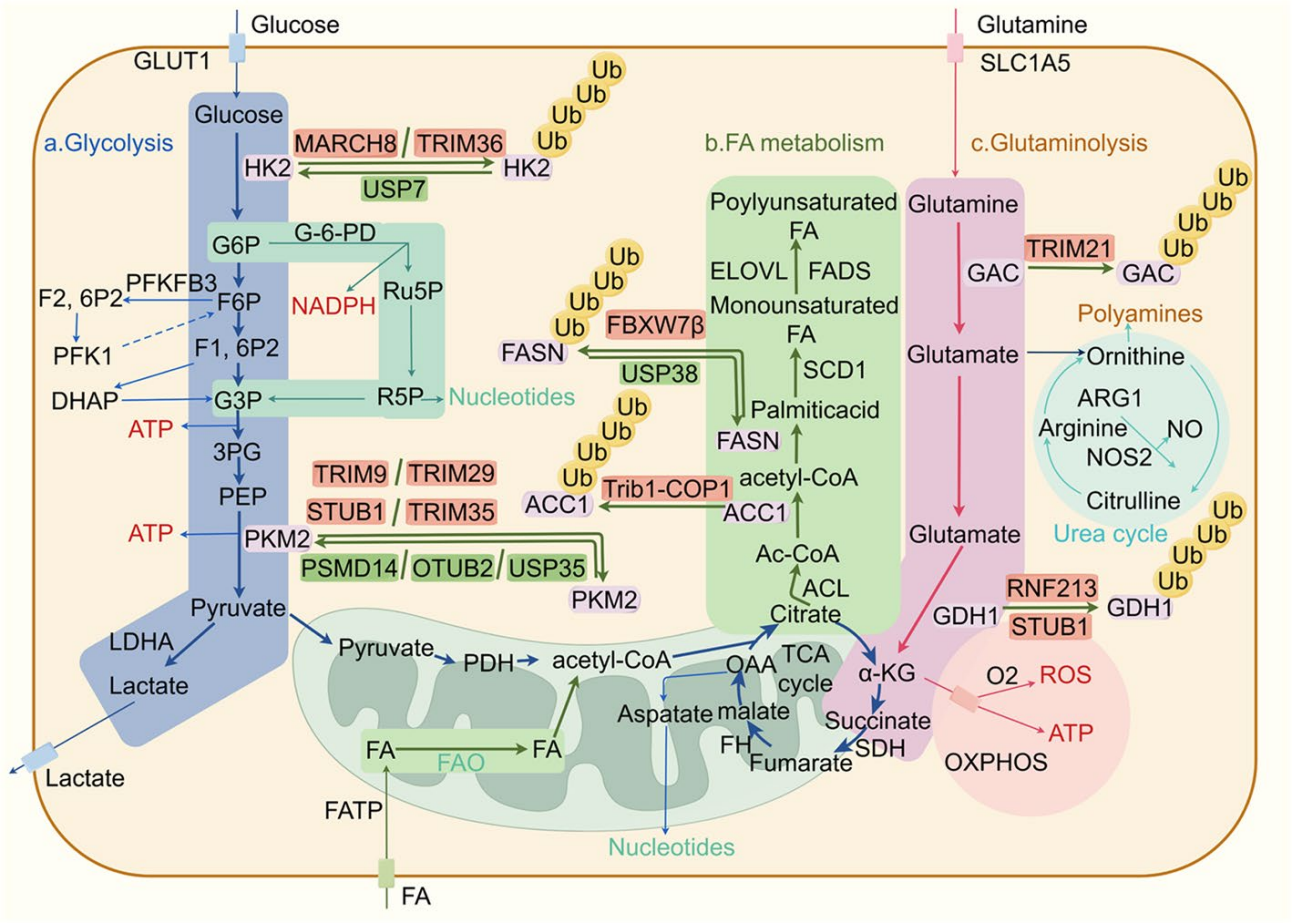
The ubiquitin–proteasome system regulates tumor metabolism in several pathways. **a** Glycolysis: Hexokinase 2 (HK2) can be ubiquitinated by E3 ligases membrane-associated RING-CH protein 8 (MARCH8) and tripartite motif protein 36 (TRIM36), a process that can be reversed by the deubiquitinase ubiquitin-specific protease 7 (USP7). Pyruvate kinase M2 (PKM2) can be ubiquitinated by E3 ligases TRIM9, TRIM29, STIP1 homology and U-box-containing protein 1 (STUB1), and TRIM35. Conversely, PKM2 can be deubiquitinated by proteasome non-ATPase regulatory subunit 14 (PSMD14), OTU domain-containing ubiquitin aldehyde-binding protein 2 (OTUB2), and USP35. **b** Fatty acid (FA) metabolism: Acetyl-CoA carboxylase 1 (ACC1) and fatty acid synthase (FASN), both involved in fatty acid metabolism, can be ubiquitinated by E3 ligases tribbles pseudokinase 1 (Trib1)-constitutive photomorphogenic 1 (COP1), and F-box and WD repeat domain containing 7β (FBXW7β), respectively. In contrast, FASN can be deubiquitinated by USP38. **c** Glutaminolysis: Glutaminase C (GAC), which catalyzes the initial step of glutamine decomposition into glutamic acid and ammonia, can be ubiquitinated by the E3 ligase TRIM21. This leads to K63-linked ubiquitination that inhibits GAC activity. Glutamate dehydrogenase (GDH), another key enzyme in glutamine catabolism, can be ubiquitinated by E3 ligases ring finger protein 213 (RNF213) and STUB1

Hexokinase 2 (HK2) phosphorylates glucose to produce glucose-6-phosphate (G-6-P), regulating glucose metabolism [330]. The E3 ligase TRIM36 ubiquitinates HK2 to inhibit the neuroendocrine differentiation (NED) of prostatic cancer (Fig. 5) [153]. Additionally, studies have shown that TRIM36 can enhance the efficacy of anti-androgen drugs in treating prostate cancer. Therefore, adding TRIM36 during androgen deprivation therapy (ADT) could be a novel therapeutic approach to better suppress castration-resistant prostatic cancer [331]. Recent research has discovered that the E3 ligase membrane-associated RING-CH protein (MARCH8) is a novel glycolysis repressor that inhibits glycolysis in colorectal cancer through the ubiquitination and degradation of HK2 (Fig. 5) [154]. However, clinical studies on MARCH8’s role in cancer metabolism are lacking, with most research focusing on its involvement in cancer cell apoptosis [332]. Future studies could explore therapeutic strategies targeting MARCH8 and its regulatory mechanisms in cancer metabolism, such as combination therapy, to enhance treatment efficacy. In turn, the circular RNA derived from ribosomal protein S19 (circRPS19) upregulates USP7 expression, leading to an increase in HK2 protein levels and the promotion of aerobic glycolysis in gastric cancer cells [165]. Therefore, targeting the circRPS19-USP7-HK2 pathway presents a promising therapeutic strategy for treating gastric cancer. PKM2 converts phosphoenolpyruvate (PEP) to pyruvate in the last step of glycolysis. On the one hand, several E3 ligases within the TRIM family, including TRIM9, TRIM29, and TRIM35, have been found to ubiquitinate PKM2 in tumor cells (Fig. 5) [157–159]. On the other hand, deubiquitinases such as OTUB2, proteasome non-ATPase regulatory subunit 14 (PSMD14) and USP35 enhance the activity and stability of PKM2, thereby promoting glycolysis in tumor cells (Fig. 5) [11, 169, 170]. Additionally, some lncRNAs including the lncRNA LINC01554 and lncRNA UCA1 have been proven to facilitate the ubiquitination of PKM2, thereby suppressing the Warburg effect [333, 334]. Therefore, developing targeted inhibitors for these enzymes and combining them with traditional chemotherapy could enhance treatment efficacy by addressing multiple aspects of tumor metabolism and growth. In conclusion, ubiquitination plays a crucial role in glucose metabolism by regulating key enzymes. This posttranslational modification influences the activity and stability of enzymes involved in glucose metabolism, impacting the overall cellular energy balance in cancer.

#### Fatty acid metabolism

Fatty acid metabolism is regulated by three rate-limiting enzymes: ATP-citrate lyase (ACLY), acetyl-CoA carboxylase (ACC), and fatty acid synthase (FASN) [335, 336]. Peroxisome proliferator-activated receptor γ (PPARγ) is a pivotal regulatory protein that is highly expressed in adipocytes and participates in lipid uptake, synthesis, and storage [337]. USP22 upregulates the expression of ACC and ACLY by promoting the K48-linked ubiquitination of PPARγ, ultimately fostering lipid accumulation and tumorigenesis in hepatocellular carcinoma cells [172]. Furthermore, its overexpression has been linked to a poor prognosis for a number of cancers [338–340]. This suggests that USP22 could serve as a novel tumor marker for cancer prognosis. The E3 ligase Trib1-COP1 complex targets ACC1 for ubiquitination and degradation, inhibiting leukemia-initiating cells and promoting myeloid differentiation in AML, protecting against leukemia-related mortality and impeding the progression of acute myeloid leukemia (Fig. 5) [162]. Hence, identifying a ubiquitination enzyme that counteracts the ubiquitination of Trib1-COP1 is crucial for targeting the fatty acid metabolism pathway for cancer treatment. Another study found that CSN6 can antagonize the activity of the E3 ligase FBXW7β, preventing FBXW7β-mediated ubiquitination and degradation of FASN, thereby positively regulating lipogenesis in colorectal cancer (Fig. 5) [163]. Importantly, the study demonstrated that combining cetuximab with orlistat can inhibit the growth of CSN6-high patient-derived xenograft (PDX) tumors. These findings hold key prognostic and therapeutic significance for colorectal cancer patients [163]. On the other hand, USP38 can deubiquitinate and stabilize FASN in gastric cancer, increasing triglyceride production and promoting growth and migration in gastric cancer cells [204]. In addition, orilistat, an inhibitor of USP38, can reverse the phenotype of USP38 overexpressed gastric cancer cells [204]. Therefore, targeting FASN with USP38 inhibitors can be used as a potential treatment for gastric cancer patients with high expression of USP38. Moreover, previous reports have indicated that FASN serves as a substrate for USP14 in hepatocytes, but other reports suggest that FASN levels in cancer cells are not considerably impacted by the USP14 inhibitor IU1, suggesting that FASN may not be a direct substrate of USP14 in cancer cells [341, 342]. Given the above, further investigations are needed to clarify the functions of the ubiquitination and deubiquitination of pivotal enzymes in fatty acid metabolism.

#### Amino acid metabolism

Amino acid metabolism in cancer is influenced by ubiquitination and deubiquitination. Glutamate dehydrogenase (GDH) catalyzes the deamination of glutamic acid and has two subtypes, GDH1 and GDH2, of which GDH1 is mainly degraded by the ubiquitin–proteasome pathway [343]. The E3 ligase RNF213 mediates GDH1 degradation in kidney renal clear cell carcinoma (KIRC) (Fig. 5) [161]. Researchers found that the loss of GDH1 promotes tumor formation after amino acid deprivation by reducing α-ketoglutarate (αKG) levels and αKG-dependent lysine demethylase (KDM) activity [161]. Additionally, another study in hepatocellular carcinoma identified two GDH1 inhibitors: Quercetin and Permethylated Anigopreissin A [344]. We can hypothesize that applying these drugs to KIRC could maintain αKG levels and KDM activity, potentially preventing the progression of KIRC. The only enzyme in mammals capable of eliminating ammonia and glutamic acid and synthesizing glutamine de novo is glutamine synthetase (GS) [345]. In multiple myeloma (MM), USP15 controls the ubiquitination of GS, which is mediated by the E3 ligase complex Cul4-DDB1-CRBN-RBX1 (CRL4^CRBN^) [171]. Interestingly, immunomodulatory drug (IMiD)-resistant cells have high expression of USP15, and lenalidomide, an immunomodulatory medication, can sensitize these cells when USP15 is depleted [171]. Thus, focusing on USP15 offers a significant therapeutic potential to improve the efficacy of CRBN-based PROTAC treatments for the treatment of cancer. A preclinical study mentioned a small molecule inhibitor of USP15 (USP15-Inh) provided by Forma Therapeutics [346]. Glutaminase (GAC) catalyzes the initial step of glutamine decomposition, converting it into glutamic acid and ammonia [347]. The E3 ligase TRIM21 promotes K63-linked ubiquitination of GAC, inhibiting its activity in non-small cell lung cancer (Fig. 5) [164]. Furthermore, acetylation of Lys311 on GAC further enhances this inhibitory process, thereby suppressing non-small cell lung cancer progression and offering new insights for targeting TRIM21 in lung cancer therapy [164].

### Evading immune destruction

#### The crosstalk between the ubiquitin protein system and the TME

Tumors are closely related to the surrounding microenvironment and constantly interact with each other [348]. Ubiquitination is a common posttranslational modification that plays a vital role in regulating cellular signal transduction pathways in the TME [320]. This modification effectively stimulates antitumor immunity and modulates the balance between tumor suppressors and oncoproteins by modulating the immune response [349]. The function of the UPS is to influence the TME by directly or indirectly regulating the degradation of immune checkpoint molecules and the release of oncogenic cytokines [8]. In conclusion, TME is an important component affecting tumor growth and development. The UPS affects tumor progression by regulating the interaction between tumors and the TME (Fig. 3).

#### The role of ubiquitination in crosstalk between CAFs and tumor cells

Cancer-associated fibroblasts (CAFs) are the most common cells in the TME. CAFs regulate the activities of tumor cells and other stromal cells through direct contact and by secreting regulatory factors, especially TGF-β, IL-6, and CC-chemokine ligand 2 (CCL2). Therefore, CAFs play an essential role in tumor progression [350].

**Fig. 6:**
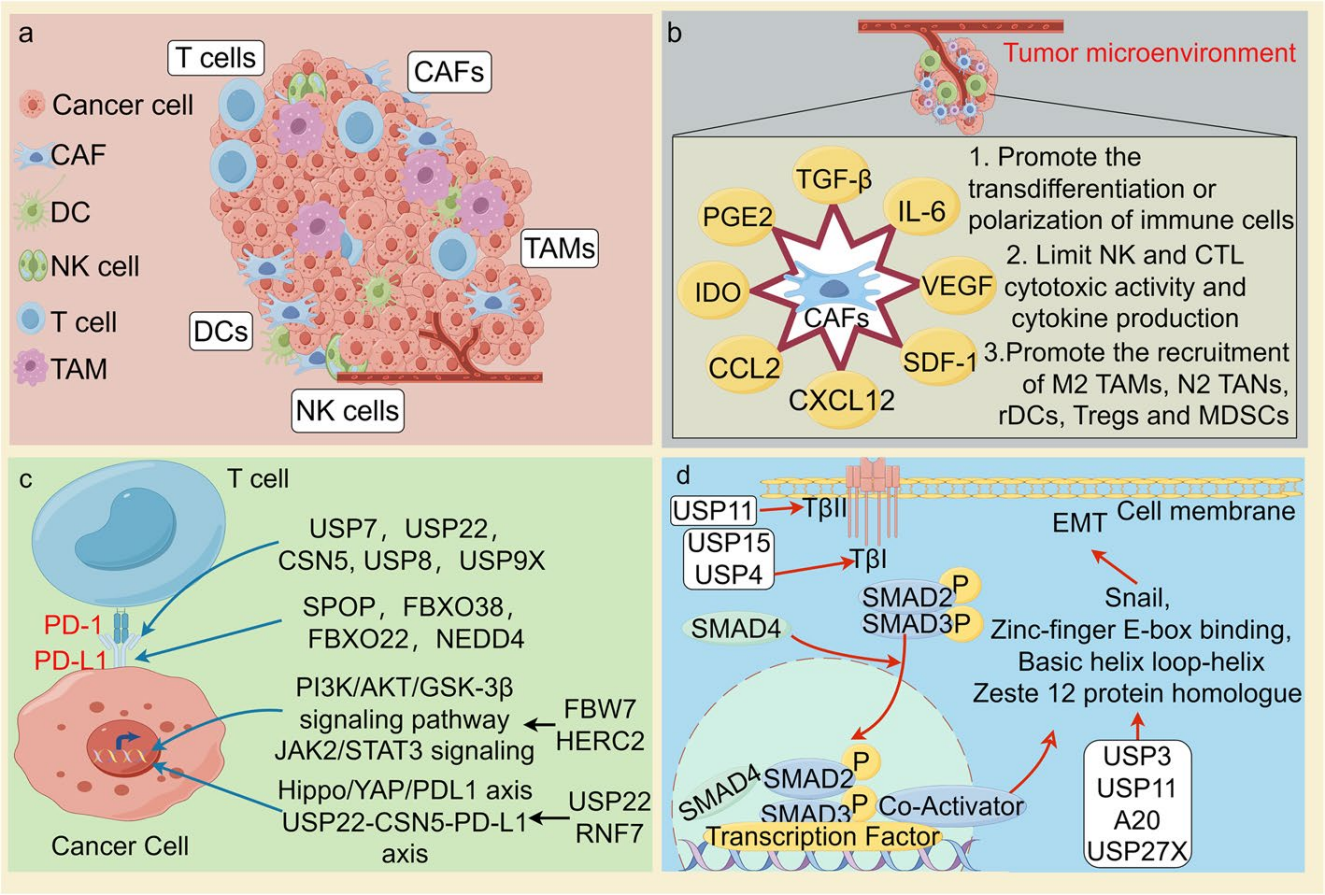
The ubiquitin–proteasome system regulates tumor immunity. **a** Components of the tumor microenvironment include cancer-associated fibroblasts (CAFs), dendritic cells (DCs), natural killer (NK) cells, tumor-associated macrophages (TAMs), and T lymphocytes. **b** CAFs secrete a variety of chemokines, cytokines, and other effector molecules, such as transforming growth factor-β (TGF-β), interleukin-6 (IL-6), C-X-C chemokine ligand 12 (CXCL12), C–C chemokine ligand 2 (CCL2), stromal cell-derived factor 1 (SDF-1), vascular endothelial growth factor (VEGF), indoleamine 2,3-dioxygenase (IDO), and prostaglandin E2 (PGE2). These molecules regulate the function of immune cell populations in the TME, mediated by immune cells to inhibit immune responses. **c** E3 ligases and deubiquitinases that directly target PD-L1. E3 ligases USP7, USP22, CSN5, USP8, and USP9X stabilize PD-L1. Conversely, deubiquiting enzymes SPOP, FBXO38, FBXO22, and NEDD4 degrade PD-L1 through ubiquitination. The following list comprises the E3 ligases and deubiquitinating enzymes involved in processes that affect PD-L1 transcription. FBW7 and RNF31 inhibit PD-L1 transcription through the PI3K/AKT/GSK-3β signaling pathway and the Hippo/YAP/PD-L1 axis. USP22 and HERC2 promote PD-L1 transcription through the USP22-CSN5-PD-L1 axis and the JAK2/STAT3 signaling pathway, respectively. **d** The involvement of the ubiquitin–proteasome system in the TGF-β signaling pathway includes USP11 acting on the TGF-β type II receptor, USP15, and USP4 acting on the TGF-β type I receptor, and USP3, USP11, A20, and USP27X acting on EMT transcription factors

TGF-β plays a vital role in the epithelial mesenchymal transition (EMT), promoting the transition of epithelial cells to motile mesenchymal cells and thereby promoting the migration and invasion of tumor cells (Fig. 6) [351]. The USP family is involved in regulating the TGF-β-induced EMT. According to recent literature, USP3, USP4, USP11, USP15, and USP26 positively regulated TGF-β signaling in various cancer types [194, 197–199]. For example, USP11 regulates TGF-β-induced plasticity and promotes breast cancer metastasis by stabilizing TGF-βRII [196]. High expression of USP11 was found in gastric cancer patients’ tumor samples, and its upregulation promoted gastric cancer tumor growth and metastasis. Interestingly, suppression of USP11 enhanced the sensitivity of GC cells to chemotherapy [352]. Additionally, in human basal-like basal cells, overexpression of the ubiquitin-editing enzyme A20 amplifies the TGF-β1-induced epithelial-mesenchymal transition by enhancing the polyubiquitination of Snail1. Knockdown of A20 reduces cancer metastasis in mouse xenograft tumors and an orthotopic breast cancer model, suggesting that the polymononubiquitination of A20 and Snail1 plays a key role in the metastasis process [184]. In addition, the DUB USP27X is regulated by TGF-β during the EMT and maintains the stability of Snail1 in breast cancer and prostate cancer. Inhibition of USP27X leads to the destabilization of Snail1, inhibits the EMT process, and enhances the sensitivity of tumor cells to chemotherapy [200].

#### The role of the ubiquitin protein system in immune checkpoints

Increasing evidence has shown that the UPS plays an important role in immune checkpoints [353]. The primary focus is on the role of the UPS in the PD-1/PD-L1 pathway [8]. These findings suggest that the UPS is a novel approach for enhancing antitumor immunity. E3 ligases and deubiquitinases are pivotal in modulating the stability of PD-1 and PD-L1 [176]. SPOP proteins regulate various cancer-related substrates and play a crucial role in mediating PD-L1 degradation [354]. Research has found that CDK4/6 increases PD-L1 protein levels by inhibiting the phosphorylation of SPOP mediated by cyclin D-CDK4. The combination of CDK4/6 inhibitors with anti-PD-1 immunotherapy has been shown to reduce tumors and significantly improve overall survival rates in mouse tumor models [173]. Additionally, as a subunit of the SCF E3 ligase complex, the F-box protein plays various roles in human tumors, including mediating the ubiquitination of PD-1 [176, 177]. One experiment showed that FBXO22 could activate the ubiquitination of PD-L1 to increase the sensitivity of non-small cell lung cancer cells to DNA damage, with CDK5 acting as an upstream regulator of FBXO22. Research suggests that combining CDK5 inhibitors with immune checkpoint inhibitors enhances the efficacy of immune checkpoint blockade therapy [178]. In addition, USP22, and USP9X stabilize PD-L1 through deubiquitination, promoting cancer development and migration [187, 193, 355]. Studies also show a positive correlation between PD-L1 and USP12 levels, with USP12 knockout desensitizing mouse lung tumor cells to anti-PD-1 therapy [192]. Additionally, USP14 knockout or inhibition enhances cell response to PD-1 inhibitors [191]. UPS also plays a vital role in cytotoxic T-lymphocyte-associated antigen 4 (CTLA4) [185]. TRAF6 is involved in CD8 + T-cell-mediated antitumor immunity by promoting the ubiquitination and degradation of CTLA-4 [185]. The role of the UPS in immune checkpoints, particularly the PD-1/PD-L1 pathway, suggests a potential for novel therapeutic strategies aimed at enhancing antitumor immunity.

### Unlocking phenotypic plasticity

During organ development, cells organize into tissues and undergo terminal differentiation, often leading to irreversible growth cessation. This process acts as a barrier to the sustained proliferation needed for tumor formation. Phenotypic plasticity can lead to changes such as dedifferentiation, blocked terminal differentiation, and transdifferentiation [356]. Subsequently, the impact of ubiquitination on surface plasticity was explored through an examination of its effects on dedifferentiation and transdifferentiation, along with a discussion of the processes of ubiquitination and deubiquitination.

The first step is dedifferentiation from the mature state to the progenitor cell state. Dedifferentiated liposarcoma (DDLPS) is an invasive adipose cell carcinoma. Characterized by a low tumor mutation burden and frequent chromosomal structural abnormalities, DDLPS often exhibits amplification of the MDM2 gene. MDM2, an E3 ligase, is responsible for targeting the degradation of p53. The overexpression of MDM2 in human cancers has been associated with a poor prognosis [202]. Targeting MDM2 is a promising therapeutic strategy, as demonstrated by the growing number of MDM2 inhibitors, such as RG7112, AMG-232, and APG-115, undergoing clinical trials [357]. Second, transdifferentiation refers to the transformation of tissue cells into cells of different lineages. The ubiquitin proteasome system is involved in the transdifferentiation process of specific cancers. The transformation of adventitial fibroblasts (AFs) into myofibroblasts is pivotal in the vascular restructuring observed in conditions such as atherosclerosis, restenosis, and aortic aneurysm. Notably, NADPH oxidase 4 (Nox4) undergoes ubiquitination through direct engagement with the ubiquitin-specific protease domain of CYLD. Elevated levels of CYLD and Nox4 in the adventitia due to hyperhomocysteinemia significantly enhance AF transformation, exacerbating CaPO_4_-induced abdominal aortic aneurysm progression in mice [203]. Furthermore, neuroendocrine prostate cancer represents a deadly subtype of prostatic cancer that is distinguished by the attenuation of AR signaling during neuroendocrine transdifferentiation. This alteration ultimately contributes to the development of drug resistance to AR-targeted therapies. Researchers have shown that the differentially expressed gene Rac GTPase-activating protein 1 (RACGAP1) is involved in the NE transdifferentiation of prostatic cancer. The underlying mechanism is that RACGAP1 promotes the neuroendocrine transdifferentiation of prostatic cancer by stabilizing the expression of EZH2 in the ubiquitin proteasome pathway [358].

### Nonmutational epigenetic reprogramming

In recent years, as research on chromatin and histones has deepened, epigenetics has gradually emerged. Epigenetics encompasses diverse modifications in gene expression that occur without modifying the gene’s DNA sequence, resulting in hereditary changes in the gene’s function [359, 360]. Epigenetic phenomena include DNA methylation, histone modification, chromatin remodeling, and noncoding RNAs [361, 362]. Studies on epigenetic phenomena related to ubiquitination and deubiquitination include DNA methylation, histone modification, and chromatin remodeling (Fig. 3).

#### DNA methylation

DNA hypermethylation of the promoter at the cytosine-phosphate-guanine (CpG) sequence is a clearly defined epigenetic marker in all human tumor types and leads to the silencing of tumor suppressor genes (TSGs) and other genes associated with cancer, thus giving precancerous cells a selective advantage [363, 364]. Recent research on ubiquitination and deubiquitination in DNA methylation focuses on DNA methyltransferases (DNMTs). The E3 ligase RNF180 ubiquitinates DNMT1, significantly reducing PCDH10 methylation levels and increasing its expression in gastric cancer. Furthermore, the positive co-expression of RNF180 and PCDH10 is associated with a favorable clinical prognosis in gastric cancer patients, suggesting that PCDH10 and RNF180 could be potential biomarkers for gastric cancer diagnosis [206]. A similar study revealed the RNF180/DNMT3A/ADAMTS9 axis in gastric cancer. ADAMTS9 significantly inhibits cell viability and motility both in vitro and in vivo. RNF180 ubiquitinates DNMT3A, markedly reducing ADAMTS9 methylation levels and increasing its expression in gastric cancer [207]. Therefore, finding an inhibitor that targets RNF180 could potentially inhibit the progression of gastric cancer from multiple angles. Fructose-1,6-bisphosphatase 1 (FBP1) is an enzyme that catalyzes a key step in gluconeogenesis, converting fructose-1,6-bisphosphate to fructose-6-phosphate [365]. Nuclear FBP1 has been found to interact with DNMT1 and to recruit PARP1 to chromatin, enhancing the sensitivity of pancreatic cancer to the poly ADP-ribose polymerase (PARP) inhibitor Olaparib [211]. Significantly, USP7 can reverse this by deubiquitinating FBP1, thereby inhibiting this interaction. Consequently, USP7 inhibitors enhanced the anti-tumor effects of PARP inhibitors in an FBP1-dependent manner. Therefore, combining USP7 inhibitors with PARP inhibitors might yield a more potent anti-tumor response than using PARP inhibitors alone, potentially offering a more effective treatment approach for PC [211].

#### Histone ubiquitination

Histone octamer is composed of H2A, H2B, H3, and H4, which are connected with DNA to form nucleosomes [366]. Histone ubiquitination contributes to proper DSB repair and plays a significant role in the interaction with transcription and replication [367]. The E3 ligase RNF40 can interact with the E3 ligase complex DNA binding protein 1 (DDB1)-Cullin 4a (CUL4A), inhibiting the ubiquitination of H2A by DDB1-CUL4A [208]. Notably, RNA demethylase ALKB Homolog 5 (ALKBH5)-mediated m6A deficiency in osteosarcoma leads to increased expression of USP22 and RNF40, suppressing H2A ubiquitination and promoting gene expression related to replication and DNA repair, driving osteosarcoma progression [208]. Therapeutic strategies could focus on creating modulators to enhance or mimic ALKBH5 activity, targeting the overexpression of USP22 and RNF40, offering a new potential treatment for cancers with m6A abnormalities. Cyclin B1, encoded by the CCNB1 gene, is regulated by H2B ubiquitination at its promoter by the E2 enzyme radiation sensitive 6 (RAD6) in esophageal squamous cell carcinoma (ESCC), affecting ESCC cell proliferation [205, 368]. Additionally, a novel RAD6 selective small molecule inhibitor targeting the catalytic site of RAD6 (SMI#9) enhanced the sensitivity of cancer cells resistant to cisplatin or oxaliplatin in triple-negative breast cancer and colorectal cancer [369]. Using SMI#9 could provide a strategy to overcome drug resistance in chemotherapy, offering a promising avenue for enhancing the effectiveness of existing cancer treatments in various resistant tumors. Therefore, specific human proteins can affect histone ubiquitination by regulating E3 ligases or E2 ubiquitin binding enzymes, impacting the cell cycle and cancer development. Refer to section II.A for additional details on histone ubiquitination.

#### Chromatin remodeling

Nucleosomes, linker histones, and nonhistones undergo further assembly into highly organized chromatin structures, restricting access to DNA [370]. The main subfamilies of chromatin remodeling complexes are switch/sucrose nonfermentable (SWI/SNF), imitation SWI (ISWI), chromodomain-helicase DNA-binding protein (CHD), and inositol-requiring mutant 80 (INO80) [371, 372]. The SWI/SNF complex was the first remodeling complex discovered [373]. In prostate cancer, OTUD6A deubiquitinates the SWI/SNF ATPase subunit Brg1, promoting cancer progression [212]. AT-rich interactive domain protein 1A (ARID1A), a SWI/SNF complex component, acts as a tumor suppressor [374]. In gastric cancer and hepatocellular carcinoma, the E3 ubiquitin ligase complex SCF promotes degradation of ARID1A, which is triggered by ATM activation due to DNA damage in gastric cancer and by mTORC1 activation in hepatocellular carcinoma [186, 209]. Some progress has been made in the clinical application of targeted anticancer therapies focusing on SCF complexes, particularly through inhibitors of cullin neddylation and Skp2, such as MLN4924 and Erioflorin [375, 376]. During normal DNA synthesis, BAP1 stabilizes INO80 through deubiquitination. Additionally, BAP1 recruits INO80 to replication forks by interacting with H2Aub, thereby facilitating fork progression. This process underscores the molecular basis of BAP1’s tumor suppressor function [377]. Additionally, recent research indicates that the E3 ligase CHIP also can stabilize INO80 through nondegradable ubiquitination. Therefore, CHIP and BAP1 collaborate to regulate the ubiquitination of INO80, thereby facilitating DNA replication [210]. These findings are noteworthy for the investigation of chromatin remodeling in cancer.

### Polymorphic microbiomes

Increasing evidence suggests that the polymorphic variability of the microbiome between individuals impacts cancer phenotypes, and distinct characteristic microbiota have been recognized in different tumors [378, 379]. Micromonas infection can enhance the proliferation and inflammatory response of colorectal cancer cells, and *Parvimonas micra (P. micra)* was found to affect protein expression in colorectal cancer intestinal epithelial cells. The upregulation of proteasome β4 (PSMB4) protein indicates the critical role of the ubiquitin proteasome pathway in colorectal cancer [213]. In addition, propionate, a microbial metabolite, was shown to target euchromatic histone lysine methyltransferase 2 (EHMT2) by coordinating proteasomal degradation through the upregulation of HECT domain E3 ubiquitin protein ligase 2 (HECTD2). Propionate treatment initially increases the expression of HECTD2, which then facilitates the proteasomal degradation of EHMT2 through posttranslational modification. EHMT2, through H3K9 dimethylation, forms heterochromatin structures and negatively regulates tumor necrosis factor alpha-induced protein 1 (TNFAIP1) [214]. Consequently, the degradation of EHMT2 reduces H3K9 dimethylation in the TNFAIP1 promoter region, leading to the upregulation of TNFAIP1 and apoptosis of colorectal cancer cells [214]. Furthermore, treatment of colorectal cancer cells with *Clostridium butyricum* (*C. butyricum*) decreased MYC-mediated resistance to 5-FU and enhanced the effectiveness of anti-PD-1 immunotherapy [380]. In summary, the microbiota and its metabolites can regulate tumor development and therapeutic efficacy by affecting the UPS. These experiments suggest potential therapeutic strategies and highlight the importance of microbiota mechanisms of action in cancer research.

### Senescent cells

Senescence is an irreversible state of cell cycle arrest that occurs when cells respond to various stress factors, including DNA damage or activation of oncogenes [381]. While senescence can exert a tumor suppressive effect by preventing the proliferation of damaged or mutated cells, it also has potential impacts on the development and progression of cancer [7]. Senescence in cancer may rely on oncogene-induced senescence (OIS) and tumor suppressor gene loss-induced senescence (TIS). The P16^INK4A^-RB and p53-p21-RB pathways are crucial mechanisms for initiating and maintaining growth arrest [381, 382]. Melanoma is a malignant tumor that originates from melanocytes in the skin and is rich in senescent cells. In melanoma, overexpression of mutant BRAF promotes excessive proliferation of melanocytes, inducing the expression of P16^INK4A^, which subsequently inhibits the activity of CDK4 and CDK6, leading to hypophosphorylation of RB, cell cycle arrest, and thus inhibiting cancer development [383]. In a study, primary mouse and human cells lacking BRCA2 exhibited senescence characteristics, which reversed upon the loss of ARF [98]. This may be due to the ability of ARF to inhibit the activity of E3 ligase MDM2, thus enabling normal p53 function. Targeting the interaction between MDM2 or ARF-MDM2 using small molecule inhibitors may help restore p53 function in the impaired p53 signaling pathway, promoting senescence or apoptosis in cancer cells [384]. ALRN-6924 reactivates p53 function by inhibiting two proteins, MDM2 and MDMX, subsequently inhibiting tumor cell growth [384]. Compounds like ALRN-6924, which induce inflammatory responses and reduce immune evasion in the tumor microenvironment, could be effectively combined with immunotherapies, especially in melanomas rich in senescent cells [384]. Exploring the interplay between ubiquitination, deubiquitination, and cellular senescence opens promising avenues for developing targeted therapies that can selectively modulate cell fate in cancer.

## Cancer treatment strategies

### Proteinase inhibitors

**Fig. 7:**
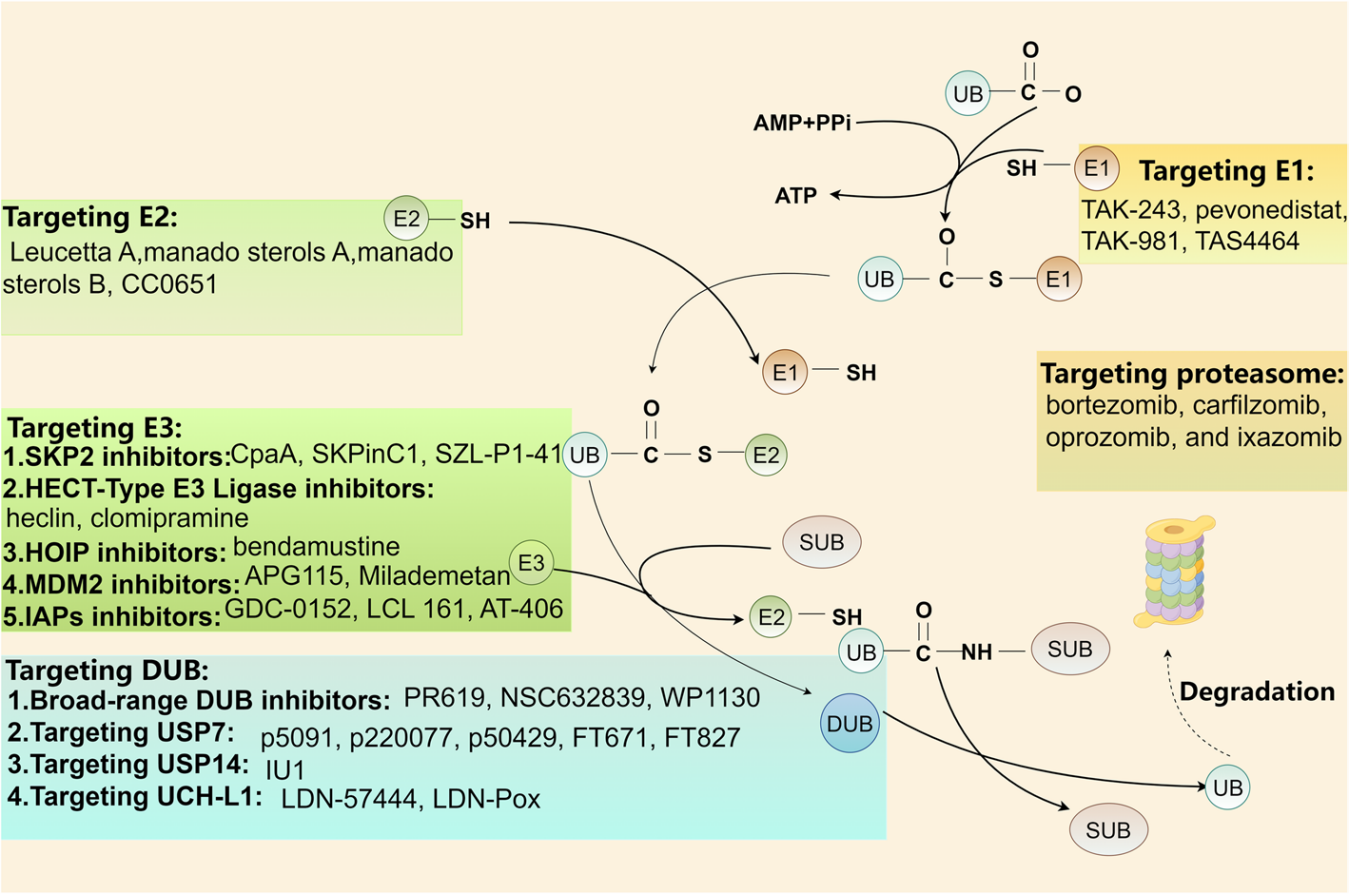
Schematic diagram of ubiquitin–proteasome system regulating proteins and its corresponding treatment strategies. Drugs targeting proteasome: bortezomib, carfilzomib, oprozomib and ixazomib. Drugs targeting El enzyme: TAK-243, pevonedistat, TAK-981 andTAS4464. Drugs targeting E2 enzyme: Leucetta A, manado sterols A, manado sterols B andCC0651. Drugs targeting E3 ligase: S-phase kinase-associated protein 2 (SKP2) inhibitors and homologous to the E6AP C-terminus (HECT)-type E3 ligase inhibitors, HOlL-1 interacting protein (HOIP) inhibitors, mouse double minute 2 (MDM2) inhibitors and IAPs inhibitors. Drugs targeting deubiquitinase (DUB): Broad-range DUB inhibitors, inhibitors targeting USP7, inhibitors targeting USP14, and inhibitors targeting UCH-L1

The proteasome is a highly anticipated target in cancer therapy. Proteasome inhibitors (PIs), like bortezomib, carfilzomib, oprozomib, and ixazomib, have been successfully developed for clinical treatment and have shown good efficacy (Fig. 7) [184, 385, 386].

Bortezomib is used to treat patients with MM by inhibiting the chymotrypsin-like activity of the 26S proteasome [387, 388]. Although its use alone may cause a wide range of unintended side effects, its toxicity can be reduced when used in combination with pomalidomide and dexamethasone [389, 390]. In addition, the FDA has approved bortezomib for treating mantle cell lymphoma [391]. Currently, bortezomib is in clinical trials for treating other types of cancer, such as autoimmune hemolysis and COAD [392, 393].

Carfilzomib is a tetrapeptide epoxide that selectively binds to the 26S proteasome and inhibits protease activity. It was approved by the FDA in 2012 for the treatment of multiple myeloma [394, 395]. Carfilzomib is undergoing clinical trials for various cancers, including renal cell carcinoma, lymphoma, acute myeloid leukemia, lymphocytic leukemia, and small-cell lung cancer.

Oprozomib is a derivative of carfilzomib designed to have better oral bioavailability. Oprozomib has shown similar antitumor activity and efficacy to carfilzomib in the treatment of multiple myeloma. Therefore, it can be used for the treatment of multiple myeloma resistant to bortezomib, dexamethasone, or lenalidomide [396]. Oprozomib induces apoptosis by upregulating the proapoptotic proteins Bcl-2 interacting killer (BIK) and MCL-1. It can be used to treat solid tumors [397]. However, oprozomib has high gastrointestinal toxicity and unstable pharmacokinetics.

Ixazomib was used to inhibit the activity of the 20S proteasome [398, 399] and its antitumor effect is superior to bortezomib [399]. Ixazomib combined with lenalidomide/dexamethasone significantly improves the survival of patients with myeloma [400]. Ixazomib has completed phase I clinical trials for glioblastoma multiforme (GBM) and phase II clinical trials for malignant myeloid and lymphatic blood cancers. The patients who exhibited resistance to bortezomib demonstrated a favorable response to isazomil. In addition to these inhibitors, other proteasome inhibitors, such as marizomib and delanzomib, are also undergoing clinical trials. For example, some studies have tested the effects of malizomil on glioblastoma through its ability to cross the blood brain barrier [395].

### E1 and E2 inhibitors

The E1 enzyme functions to activate ubiquitin and transfer it to the E2 enzyme [1]. Adenosine 3', 5' monophosphate (AMP) is tightly bound to the E1 enzyme in the catalytic cascade of ubiquitin activation, and some AMP-related drugs can act as E1 inhibitors. The major sodium adenosulfonate E1 inhibitors include perazone, TAK-243, ML-792, TAK-981, acetyl-DL-carnitine, ABPA3, and ABP1 (Fig. 7) [401]. Among the E1 inhibitors in clinical trials, the only one with published clinical trial data is pevonedistat. Combination therapy with risperidone, such as risperidone and azacitidine, has shown more promising efficacy in the treatment of AML patients, and carboplatin and paclitaxel have shown better clinical benefits in the treatment of advanced solid tumors. In patients with AML with TP53 mutations, the composite CR/PR rate was 80% with pevonedistat and azacitidine combination therapy [402, 403].

E2 enzymes primarily facilitate the binding of ubiquitin to substrates. Current efforts are focused on identifying inhibitors that disrupt the interaction between E1 enzymes and E2 enzymes or between E2 enzymes and E3 ligases. For instance, Leucetta A has been shown to inhibit the interaction between ubiquitin-conjugating enzyme 13 (UBC13) and ubiquitin-like protein one activating enzyme (UEV1A), thereby preventing complex formation [404]. Alternatively, manadosterols A and B, isolated from the sponge *Lissodendoryx fibrosa*, target the same molecular interaction as Ubc13-Uev1A [405]. In addition, the E2 enzyme Cdc34 inhibitor CC0651 blocks the ubiquitination and degradation of p27, thereby inhibiting tumor cell proliferation [406].

### E3 inhibitors

The E3 ligase interacts with the ubiquitin-activating enzyme E1 and the ubiquitin-conjugating enzyme E2 to complete the ubiquitination process [407]. Drugs targeting E3 ligases play an essential role in cancer therapy by linking ubiquitin to specific protein amino acids. Clinical research information on these inhibitors has been obtained from https://clinicaltrials.gov/ and https://pubchem.ncbi.nlm.nih.gov/, as listed in Table 2.

**Table 2:** Summary of pharmacological strategies direct targeting the ubiquitin proteasome system for cancer therapy in clinical trials (information was obtained from https://www.clinicaltrials.gov/)

Identifier	Phase	Drug	Target	Cancer	Treatment	Status	References
NCT02045095	Phase 1	Uba1	Uae	Solid tumors	Monotherapy	Terminated	NA
NCT04074330	Phase 1/phase 2	TAK-981	Sae	Lymphoma	Combination with rituximab	Completed	[408]
NCT04065555	Early phase 1	TAK-981	Sae	Head and neck cancer	Monotherapy and combination	Completed	[409]
NCT03770260	Phase 1	Pevonedistat	NEDD8	Multiple myeloma	Combination with ixazomib	Completed	NA
NCT04712942	Phase 2	Pevonedistat	NEDD8	Myeloid leukemia	Combination with azacitidine	Completed	NA
NCT03319537	Phase 1/phase 2	Pevonedistat	NEDD8	Mesothelioma	Combination with standard chemotherapy,	Completed	NA
NCT03330106	Phase 1	Pevonedistat	NEDD8	Solid neoplasm	Combination with standard chemotherapy	Completed	[410]
NCT03814005	Phase 1	Pevonedistat	NEDD8	Myeloid leukemia	Combination with standard chemotherapy	Completed	NA
NCT03349281	Phase 1	Pevonedistat	NEDD8	Lymphoblastic leukemia	Combination with vxld	Completed	NA
NCT02610777	Phase 2	Pevonedistat	NEDD8	Myeloid leukemia	Combination with azacitidine	Completed	[411]
NCT02782468	Phase 1	Pevonedistat	NEDD8	Myeloid leukemia	Combination with azacitidine	Completed	[412]
NCT03486314	Phase 1	Pevonedistat	NEDD8	Advanced solid neoplasm	Monotherapy	Completed	[413]
NCT03459859	Phase 1	Pevonedistat	NEDD8	Myeloid leukemia	Combination with low dose cytarabine	Completed	NA
Nct01862328	Phase 1	Pevonedistat	NEDD8	Solid tumors	Combination	Completed	[402]
NCT01814826	Phase 1	Pevonedistat	NEDD8	Myelogenous leukemia	Combination with azacitidine	Completed	[403]
NCT03057366	Phase 1	Pevonedistat	NEDD8	Advanced solid tumors	Monotherapy	Completed	[414]
NCT02122770	Phase 1	Pevonedistat	NEDD8	Advanced solid tumors	Monotherapy	Completed	[415]
NCT00911066	Phase 1	Pevonedistat	NEDD8	Myeloid leukemia	Monotherapy	Completed	[416]
NCT00722488	Phase 1	Pevonedistat	NEDD8	Multiple myeloma	Monotherapy	Completed	[417]
NCT00677170	Phase 1	Pevonedistat	NEDD8	Nonhematologic malignancies	Monotherapy	Completed	[418]
NCT01011530	Phase 1	Pevonedistat	NEDD8	Metastatic melanoma	Monotherapy	Completed	[419]
NCT02935907	Phase 1	APG115	MDM2	Solid tumor or lymphoma	Monotherapy	Completed	NA
NCT01877382	Phase 1	Milademetan	MDM2	Advanced solid tumor	Monotherapy	Completed	[420]
NCT03671564	Phase 1	Milademetan	MDM2	Myeloid leukemia	Monotherapy	Completed	[421]
NCT03614455	Early phase 1	Milademetan	MDM2	Pharmacokinetics	Combination	Completed	[422]
NCT02890069	Phase 1	Siremadlin	MDM2	Colorectal cancer	Combination	Completed	NA
NCT02143635	Phase 1	Siremadlin	MDM2	Solid and hematological tumors	Monotherapy	Completed	[423]
NCT02343172	Phase 1	Siremadlin	MDM2	Liposarcoma	Combination with lee011	Completed	NA
NCT01723020	Phase 1	AMG 232	MDM2	Malignancy	Monotherapy	Completed	[241]
NCT02110355	Phase 1	AMG 232	MDM2	Malignancy	Combination with trametinib and dabrafenib	Completed	[424]
NCT02016729	Phase 1	AMG 232	MDM2	Malignancy	Combination with trametinib	Completed	[425]
NCT01677780	Phase 1	RG7112	MDM2	Myelogenous leukemia	Monotherapy	Completed	NA
NCT00559533	Phase 1	RG7112	MDM2	Neoplasms	Monotherapy	Completed	NA
NCT00623870	Phase 1	RG7112	MDM2	Hematologic neoplasms	Monotherapy	Completed	NA
NCT01143740	Phase 1	RG7112	MDM2	Sarcoma	Monotherapy	Completed	NA
NCT01164033	Phase 1	RG7112	MDM2	Neoplasms	Monotherapy	Completed	[426]
NCT01605526	Phase 1	RG7112	MDM2	Sarcoma	Combination with doxorubicin	Completed	NA
NCT01636479	Phase 1	SAR405838	MDM2	Neoplasm malignant	Monotherapy	Completed	[427]
NCT01985191	Phase 1	SAR405838	MDM2	Neoplasm malignant	Combination with pimasertib	Completed	[428]
Nct02670044	Phase 1	RG7388	MDM2	Acute myeloid leukemia	Combination with venetoclax	Completed	[429]
NCT03362723	Phase 1	RG7388	MDM2	Solid tumors	Monotherapy	Completed	NA
NCT02828930	Phase 1	RG7388	MDM2	Solid tumors	Monotherapy	Completed	[430]
NCT01773408	Phase 1	RG7388	MDM2	Acute myeloid leukemia	Combination with cytarabine	Completed	[431]
NCT01462175	Phase 1	RG7388	MDM2	Neoplasms	Monotherapy	Completed	[432]
NCT01901172	Phase 1	RG7388	MDM2	Neoplasms	Combination with posaconazole	Completed	NA
NCT01760525	Phase 1	CGM097	MDM2	Solid tumor	Monotherapy	Completed	[433]
NCT02890069	Phase 1	LCl161	IAPs	Colorectal cancer	Combination	Completed	NA
NCT03111992	Phase 1	LCl161	IAPs	Multiple myeloma	Combination with pdr001	Completed	NA
NCT01240655	Phase 1	LCl161	IAPs	Solid tumors	Combination with paclitaxel	Completed	NA
NCT01968915	Phase 1	LCl161	IAPs	Neoplasms	Monotherapy	Completed	[434]
NCT01617668	Phase 2	LCl161	IAPs	Breast cancer	Combination with paclitaxel	Completed	NA
NCT01098838	Phase 1	LCl161	IAPs	Solid tumors	Monotherapy	Completed	[435]
NCT04122625	Phase 1/phase 2	AT-406	IAPs	Solid tumor	Combination with nivolumab	Completed	NA
NCT03871959	Phase 1	AT-406	IAPs	Adenocarcinoma of the pancreas	Combination with pembrolizumab	Completed	[436]
NCT02022098	Phase 2	AT-406	IAPs	Squamous cell carcinoma	Combination with cisplatin and radiotherapy	Completed	[437]
NCT03270176	Phase 1	AT-406	IAPs	Neoplasms	Combination with avelumab	Completed	NA
NCT01078649	Phase 1	AT-406	IAPs	Solid tumors	Monotherapy	Completed	[438]
NCT01940172	Phase 1	Tl-32711	IAPs	Ovarian cancer	Combination with conatumumab	Completed	[439]
NCT03386526	Phase 1	APG-1387	IAPs	Solid Tumors	Monotherapy	Completed	[440]

#### PROTACs

Small molecule drugs are designed to specifically target disease-related proteins using lock-and-key mechanisms. This approach relies on the presence of suitable pocket regions in the target protein structure as small molecule binding sites. PROTAC technology provides an essential platform for inducing the degradation of target proteins. The PROTAC molecule consists of two components, a ligand capable of specifically binding to the target protein and a ligand that recruits an E3 ligase to promote ubiquitination of the captured protein, leading to target protein degradation [441, 442]. ARV-110 and ARV-471 have progressed to phase II clinical trials. Much of the subsequent discussion has focused on these two pharmaceutical compounds.

ARV-110 utilizes PROTAC technology and has potential antitumor activity. ARV-110 can bind to the AR ligand recognition domain of the E3 ligase [12]. ARV-110 successfully completely degrades AR in various cell lines (DC50 < 1 nM) [389], and oral administration of ARV-110 (10 mg/kg) successfully inhibits the growth of enzalutamide-insensitive tumors in hepatocellular carcinoma patient-derived xenograft (PDX) models [390].

ARV-471 is an oral heterobifunctional molecule that uses PROTAC technology to target estrogen receptor (ER) α and has potential antitumor activity [443]. A phase I study involving patients with ER + and HER2-BC found that ARV-471 significantly reduced ER expression by up to 90% in tumor tissue. The phase I data indicated that ARV-471 performs well at any dose, substantially degrades ER, and is well tolerated. ARV-471 degrades both wild-type and mutant ER proteins. ARV-471 is undergoing a phase II clinical trial evaluating its efficacy in patients with ER + /HER2 + locally advanced and metastatic breast cancer [444, 445]. Oral ARV-471 monotherapy showed promising antitumor activity in estrogen-dependent MCF7 xenografts and a significant reduction in ER protein levels. Enhanced anticancer effects were noted when combined with the CDK4/6 inhibitor, palbociclib [446]. ARV-471 also showed a good inhibitory effect on a hormone-independent PDX model of estrogen receptor 1 (ESR1) mutants [447]. These results demonstrate the feasibility of the PROTAC approach in patients.

#### Molecular glue

Although PROTAC technology offers significant potential for drug development, the designed molecules are typically large. An alternative effective strategy involves the use of molecular glue degraders, small molecules capable of facilitating novel interactions between target proteins and E3, ultimately leading to ubiquitination-mediated degradation of the target protein [448]. Unlike PROTACs, molecular glues have better chemical properties and smaller molecular volumes (Table 3).

**Table 3:** Representative small molecules targeting protein degradation under clinical evaluation (information was obtained from https://www.clinicaltrials.gov/)

Identifier	Phase	Drug	Target	Cancer	Treatment	Status	References
NCT02372240	Phase 1/phase 2	VLX1570	UCHL5 and USP14	Multiple myeloma	Combination with dexamethasone	Terminated	[449]
NCT01049841	Phase 1	Perifosine	UCHL3	Pediatric solid tumors	Combination with temsirolimus	Completed	[450]
NCT00873457	Phase 2	Perifosine	UCHLl3	Refractory tumors	Monotherapy	Completed	[451]
NCT00391560	Phase 2	Perifosine	UCHL3	Leukemia	Monotherapy	Completed	NA
NCT00054145	Phase 2	Perifosine	UCHL3	Breast cancer	Monotherapy	Completed	[452]
NCT01097018	Phase 3	Perifosine	UCHL3	Colorectal cancer	Monotherapy	Completed	[453]
NCT05240898	Phase 1	KSQ-4279	USP1	Advanced solid tumors	Monotherapy and combination therapy	Active, not recruiting	NA
NCT04336982	Phase 1/phase 2	CC-90009	CUL4-DDB1-CRBM-RBX1 E3 complex	Acute myeloid leukemia	Combination therapy	Active, not recruiting	[13]
NCT00676910	Phase 1	JNJ-26854165	MDM2	Neoplasms	Monotherapy	Completed	[454]
NCT03041688	Phase 1	AMG-232	MDM2	Acute myeloid leukemia	Combination with decitabine and venetoclax	Recruiting	NA
NCT03654716	Phase 1	ALRN-6924	MDM2	Leukemia	Monotherapy	Completed	[455]
NCT02579824	Phase 1	DS-3032b	MDM2	Myeloma	Monotherapy	Terminated	NA
NCT02098967	Phase 1	RO6839921	MDM2	Acute myeloid leukemia	Monotherapy	Completed	[456]
NCT01462175	Phase 1	RO5503781	MDM2	Advanced malignancies except leukemia	Monotherapy	Completed	[457]
NCT03449381	Phase 1	BI907828	MDM2	Different types of advanced cancer	Monotherapy	Recruiting	NA
NCT05376800	Phase 1	BI907828	MDM2	Glioblastoma	Monotherapy	Recruiting	NA
NCT03964233	Phase 1	BI907828	MDM2	Different types of advanced cancer	Combination with ezabenlimab	Recruiting	NA
NCT05107674	Phase 1	NX-1607	Cbl-b	Advanced malignancies	Monotherapy and combination with paclitaxel	Recruiting	NA
NCT04283097	Phase 1	KPG-818	CRL4	Hematological malignancies	Monotherapy	Recruiting	NA

Progress has been made in the development of drugs containing the E1 and E2 enzymes. However, because E3 ligases can bind to target proteins more precisely and specifically, drugs that act on E3 ligases are expected to be developed [458]. CC-90009 can recruit SPT1 to the CRL4^CRBN^ E3 complex and promote the ubiquitination of GSP for proteasomal degradation [13]. Serdemetan (JNJ-26854165) is an antagonist of the human double minute 2 (HDM2) E3 ligase, that blocks p53 degradation by inhibiting the ubiquitination of HDM2. In addition, serdemetan can inhibit cholesterol transport. Clinical research on human cell lymphoma and multiple leiomyomas is underway [454, 459]. Notably, several small molecule drugs targeting MDM2 have been identified, as shown in Table 3. In addition, phase I clinical trials are currently underway for KPG-818, a potential therapeutic agent targeting Cullin-RING ligase 4 (CRL4) to treat hematological malignancies (NCT04283097) [460].

#### Other inhibitors

##### SKP2 inhibitors

CpaA blocks SKP2 assembly in SCF complexes, leading to G1/S cell cycle arrest and SCFSKP2/ p27-dependent cell death while overcoming multidrug resistance [461]. In another experiment, thiazolidinedione derivatives C1, C2, C16, and C20 were shown to target the SKP2-Cks1/p27 binding interface by selectively inhibiting p27 ubiquitination [462]. SZL-P1-41 can effectively inhibit SKP2 and enhance the sensitivity of glioma cells to temozolomide (TMZ) [463]. Dt204 reduces myeloma growth by reducing the binding of SKP2 to cullin and commd1 [464].

##### HECT-type E3 ligase inhibitors

Research indicates that heclin, a small molecule inhibitor, can alter the conformation of the HECT domain, significantly suppressing the activity of HECT-type E3 ligases and demonstrating antitumor properties [465]. In addition, a high-throughput screen identified that clomipramine, an inhibitor of the HECT ubiquitin E3 ligase ITCH, acts as an autophagy modulator to inhibit the growth of breast cancer, prostate adenocarcinoma, and bladder urothelial carcinoma cells [466]. Alternatively, a molecular model of the WW domain containing E3 ubiquitin protein ligase 2 (WWP2) inhibitor complex, which combines saturation transfer differential nuclear magnetic resonance (STD NMR), DEEP-STD NMR methods, and docking calculations, has recently been proposed to provide a method for the development of novel inhibitors [467].

##### MDM2 inhibitors

MDM2 can ubiquitinate and degrade p53 and is an ideal target for cancer therapy [468, 469]. APG115, which has a high affinity for MDM2 and significantly promotes tumor regression, is currently undergoing clinical trials for cancer therapy [470]. In addition, the MDM2 inhibitor APG-115 showed a synergistic effect with PD-1 blockade to enhance antitumor immunity within the TME [471]. APG-115 has shown potent antitumor activity in preclinical models of acute myeloid leukemia [472]. Inhibitors of MDM2 include milademetan, milademetan tosylate, siremadlin, siremadlin succinate, AMG 232, RG7112, SAR405838, RG7388, CGM097, and Nutlin-3A, which are in clinical trials to investigate their therapeutic effects on cancer [357, 473].

##### IAP inhibitors

IAP inhibitors were created by mimicking Smac/Diablo, a natural antagonist of IAPs, to induce the proteasome-dependent degradation of cIAP1, cIAP2, and X-linked IAPs [474]. Various small molecule inhibitors targeting the IAP are clinically available, as shown in Table 2 [473]. LCL161 treatment can induce an acute inflammatory response and activate phagocytes. In addition, LCL161 treatment can stimulate myeloma cells to secrete soluble factors through MΦs and induce tumor cell phagocytosis, thereby enhancing innate and adaptive immune responses and effectively stimulating antitumor immunity [475]. In another study, APG-1387 exerted dual anti-tumor effects on ciAP2-overexpressing HBV-positive hepatocellular carcinoma cells by inducing apoptosis and enhancing antitumor immunity [173]. Currently, several inhibitors of IAPs remain under evaluation in clinical trials (NCT04568265 and NCT04643405), and IAP inhibitors are promising novel effective immunomodulators for cancer treatment.

### DUB inhibitors

Ubiquitination is a dynamic process managed by DUBs, which facilitate the removal and alteration of UB or polyubiquitin chains from ubiquitinated proteins [476]. Many DUBs are involved in the cell cycle process, regulation of genomic instability, and various events in tumorigenesis [477]. As a result, numerous inhibitors of DUBs have been developed, including both broad-spectrum and targeted varieties, all of which are recognized as promising candidates for cancer therapy [478, 479].

G5 and F6 are broad-spectrum DUB inhibitors discovered through cell-based drug screening [480]. These chalcone DUB inhibitors are known for their ability to induce apoptosis in BCL-2-independent cells [480, 481]. Through active chemical proteomics, compound PR619 was suggested as a broad-spectrum DUB inhibitor [482]. NSC632839, another potent inhibitor with broad-spectrum activity against deubiquitinases, selectively targets USP2 and USP7, inducing apoptosis in cancer cells [480]. As a specific USP1 inhibitor, pimozide can block the maintenance and radiation resistance of glioma stem cells [483]. WP1130 inhibits several DUBs, including USP9X, USP5, USP14, ubiquitin carboxyl-terminal hydrolase isozyme L5 (UCHL5), and UCH37. It decreases the MCL-1 level and increases the p53 level, showing antitumor effects [484]. Betulinic acid, derived from various plants, has been recognized as a broad-spectrum DUB inhibitor, that triggers an aberrant transmembrane potential and apoptosis in cancer cells [485, 486]. Nevertheless, these inhibitors might amplify their impacts and nonspecific toxicity through diverse mechanisms, underscoring the clinical preference for specific DUB inhibitors [487].

USP7 is widely recognized as a target for drug development due to its key role in regulating p53 stability. USP7 antagonists, such as p5091 and p50429, have been developed to promote the ubiquitination and degradation of MDM2, leading to bortezomib resistance in MM cells [482, 488, 489]. Moreover, FT671 and FT827 target dynamic pockets near the USP7 catalytic center via self-inhibiting apolipoproteins [490], thereby disrupting the stability of the USP7 substrate, increasing the protein levels of p53 and its related genes, and ultimately inhibiting tumor growth [490, 491]. A series of small molecules, including HBX 19818, HBX28,258, P22077, and P50429, were shown by biochemical tests and protein mass spectrometry to have specific inhibitory effects on USP7 [489, 492]. USP14, which is related to WNT/β-catenin signal transduction [493, 494], is overexpressed in various cancer types and is positively associated with a poor prognosis [495, 496]. The inhibitor IU1 can effectively inhibit the activity of the USP14 enzyme by blocking its binding to the proteasome and enhancing its function [497].

Various new screening methods have been used to identify inhibitors and related compounds that target DUBs. For instance, high-throughput screening techniques were employed to identify selective inhibitors targeting UCH-L1, resulting in the identification of LDN-57444. This compound has been shown to trigger apoptosis in lung cell lines [498]. Furthermore, a cell-based screening methodology has been employed to identify compounds that can induce apoptosis across various tissue types, with b-AP15 serving as a prominent example [499]. B-AP15 triggers the accumulation of high molecular weight ubiquitin (Ub) complexes within cells and acts as an inhibitor of 19S regulatory particles. It selectively targets the ubiquitination activity of USP14 and other ubiquitin enzymes without impacting proteasome function [500]. In addition, b-AP15 inhibits the degradation of proteasome substrate proteins, leading to the accumulation of ubiquitin, which induces significant protein stress and mitochondrial damage [501, 502]. In various solid tumors and multiple myeloma, it may lead to tumor apoptosis through a c-MYC-NoXa-mediated pathway [500, 501, 503].

## Conclusion

In the past few decades, significant progress has been achieved in the study of the UPS. In this review, we comprehensively reviewed the research progress on the UPS regarding tumor characteristics and treatment strategies. Regarding tumor characteristics, the specific mechanisms by which ubiquitination influences cancer progression remain unclear. Therefore, future investigations must delve into the specific mechanisms involved and endeavor to elucidate the efficacy of the UPS. In terms of treatment strategies, although we have collected some clinical drug information, we also noticed that some knowledge gaps persist, especially regarding E1- and E2-targeted drugs. These findings suggest that future drug research should focus more on these aspects.

Based on the role of the UPS in cancer, potential therapeutic targets have been identified, and the corresponding inhibitors have been further studied. Proteasome inhibitors, such as bortezomib, carfilzomib, oprozomib, and ixazomib, have been approved by the FDA. They have achieved good clinical results, but their widespread application is limited by the side effects caused by the abnormal accumulation of some upstream proteins. Therefore, targeted inhibitors of E1 enzymes, E2 enzymes, E3 ligases, deubiquitinases, and other targets, including MDM2 inhibitors, IAPs inhibitors, and SKP2 inhibitors, are being investigated [473]. PROTACs and molecular glues are also being developed for the treatment of cancer. At the same time, high-throughput screening also helps researchers screen suitable inhibitors [467]. Furthermore, during tumor proliferation, the UPS may influence various oncogenic signaling cascades, concurrently dysregulating multiple pathways and thereby complicating the development of targeted therapeutic strategies. Therefore, multitarget combination therapy is a direction for future development. Finally, further exploration of UPS function and clinical studies will provide important implications for the development of new cancer treatment strategies.

## Data Availability

No datasets were generated or analysed during the current study.

## Abbreviations

ACC: Acetyl-CoA carboxylase
ACLY: ATP- citrate lyase
AF: Adventitial fibroblast
AHNAK: Neuroblast differentiation-associated protein
AIP4: Atrophin-interacting protein 4
ALDH2: Aldehyde dehydrogenase 2
ALKBH5: ALKB Homolog 5
AML: Acute myeloid leukemia
AMP: Adenosine 3', 5' monophosphate
ANLN: Anillin
APC: Anaphase-promoting complex
AR: Androgen receptor
ARF: ADP-ribosylation factor
ARID1A: AT-rich interactive domain protein 1A
ASK1: Apoptosis signal-regulating kinase 1
ATM: Ataxia telangiectasia mutated
ATR: Ataxia telangiectasia mutated Rad3-related kinase
AXIN: Axis inhibitory protein
BAP1: BRCA1 associated protein 1
BECN1: Beclin 1
BIK: BCL2 interacting killer
BRCA1: Breast cancer susceptibility gene 1
BRG1: Brahma-related gene 1
CAF: Cancer-associated fibroblast
CASTOR1: Cytosolic arginine sensor for mTORC1 subunit 1
Cbl: Casitas B-lineage lymphoma
CCF: Cytoplasmic chromatin fragments
CCL2: CC-chemokine ligand 2
CCNB1: Cyclin B1
CDH1: E-cadherin
CDK: Cyclin-dependent kinase
CDKN2A: Cyclin-dependent kinase inhibitor 2A
CHD1: Chromodomain helicase DNA binding protein 1
CHIP: C-terminus of Hsp70-interacting protein
CHK1: Cell cycle checkpoint kinase 1
CK1a: Casein kinase 1a
CLK2: CDC2-like kinase 2
COAD: Colon adenocarcinoma
CPG: Cytosine phosphate guanine
CRL4^CRBN^: Cul4-DDB1-CRBN-RBX1
CSN6: COP9 signalosome subunit 6
CtIP: CtBP-interacting protein
CTLA4: Cytotoxic T-lymphocyte-associated antigen 4
CUL3: Cullin-3
CYLD: Cylindromatosis
DAXX: Death-associated protein
DCAF1: DDB1- and CUL4-associated factor1
DDLPS: Dedifferentiated liposarcoma
DDR: DNA damage response
DDX39B: DExD-box helicase 39B
DLK1: Delta-like canonical notch ligand 1
DNMT: DNA methyltransferase
DSB: Double-strand break
DUB: Deubiquitinase
EGFR: Epidermal growth factor receptor
EHMT2: Euchromatic histone lysine methyltransferase 2
EMT: Epithelial-mesenchymal transition
ENO2: Enolase 2
ER: Estrogen receptor
ESCC: Esophageal squamous cell carcinoma
ESR1: Estrogen receptor 1
EZH2: Enhancer of zeste homolog 2
FASN: Fatty acid synthase
FBP1: Fructose -1,6- bisphosphatase 1
FBW7: F-box and WD repeat domain-containing 7
FBXL18: F-box and leucine-rich repeat protein 18
FBXO22: F-box protein 22
FBXW2: F-box and WD repeat domain containing 2
FZR1: Fizzy-related 1
G-6-PD: Glucose 6-phosphate dehydrogenase
GAC: Glutaminase C
GAK: Cyclin G-associated kinase
GBM: Glioblastoma multiforme
GCN5: General control nonderepressible-5
GDH: Glutamate dehydrogenase
GPX4: Glutathione peroxidase 4
GS: Glutamine synthetase
GSDEM: Gasdermin E
GSK3β: Glycogen synthase kinase 3β
GSPT1: G1-to-S phase transition 1
H2Aub: H2A ubiquitination
H2BK120: Lysine 120 on histone H2B
H2Bub1: Histone H2B monoubiquitination
HCF-1: Host-cell factor 1
HECT: Homologous to E6AP C-terminus
HECTD2: HECT domain E3 ubiquitin protein ligase 2
HERC1: HECT domain and RCC-1 like domain 1 gene
HIF: Hypoxia-inducible factor
HK2: Hexokinase 2
HNSC: Head and neck squamous cell carcinoma
HOIL-1L: Heme-oxidized IRP2 ubiquitin ligase 1
HOIP: HOIL-1 interacting protein
HR: Homologous recombination
HUWE1: HECT, UBA, and WWE domain containing E3 ligase 1
IAP: Inhibitor of apoptosis protein
IBRDC2: IBR domain containing 2
ICC: Intrahepatic cholangiocarcinoma
IFN: Interferon
IMiDs: Immunomodulatory drugs
INO80: INOsitol-requiring mutant 80
INSIG1: Insulin-induced gene 1
IRS-1: Insulin receptor substrate-1
ISG15: Interferon-stimulated gene 15
ISWI: Imitation SWI
ITGAV: Integrin αv
KDM: αKG-dependent lysine demethylase
KIRC: Kidney renal clear cell carcinoma
KLF5: Kruppel-like factor 5
KRT18: Keratin 18
LDHA: Lactate dehydrogenase A
LKB1: Liver kinase B1
lncRNA: Long non‐coding RNA
LPP: Lipoma preferred partner
LRR: Leucine-rich repeat domain
LUBAC: Linear ubiquitin chain assembly complex
m6A: N6-methyladenosine
MARCH: Membrane-associated RING-CH protein
MDA-7: Melanoma differentiation-associated gene-7
MDM2: Mouse double minute 2
METTL5: Methyltransferase 5, N6-adenosine
MIB2: Mind bomb homolog 2
MKK4: Mitogen-activated protein kinase kinase 4
MKRN1: Makorin ring finger protein 1
MLK3: Mixed-lineage protein kinase 3
MM: Multiple myeloma
MMP: Mitochondrial membrane potential
MOMP: Mitochondrial outer membrane permeabilization
MRE11: Meiotic recombination 11
MRN: Mre11-Rad50-Nbs1
MTSS1: Metastasis suppressor protein 1
MULAN: Mitochondrial ubiquitin ligase activator NF-κB
NBS1: Nijmegen breakage syndrome 1
NCOA4: Nuclear receptor coactivator 4
NED: Neuroendocrine differentiation
NEDD8: Neural precursor cells expressed developmentally down-regulated 8
NEMO: NF-κB essential regulator
NFAT2: Nuclear factor of activated T cell 2
NHEJ: Non-homologous end joining
NLR: NOD-like receptor
NLRP3: NOD-like receptor protein 3
NOD: Nucleotide-binding oligomerization domain
NOX4: NADPH oxidase 4
NPC: Nasopharyngeal carcinoma
NRF-1: Nuclear respiratory factor 1
NTP: Non-thermal plasma
NuRD: Nucleosome remodeling and deacetylation
OIS: Oncogene-induced senescence
OTUB2: OTU domain-containing ubiquitin aldehyde-binding protein 2
OTULIN: OTU deubiquitinase with linear linkage specificity
OXPHOS: Oxidative phosphorylation
P. micra: Parvimonas micra
PABPC1: Poly(A)-binding protein, cytoplasmic 1
PARP: Poly ADP-ribose polymerase
PCDH10: Protocadherin 10
PDAC: Pancreatic ductal adenocarcinoma
PDGFRβ: Platelet-derived growth factor receptor ß
PDK1: Phosphatidylinositol-dependent protein kinase 1
PD-L1: Programmed cell death ligand 1
PD-1: Programmed cell death 1
PDX: Patient-derived xenograft
PEBP: Phosphatidylethanolamine-binding protein
PELI1: Pellino1
PEP: Phosphoenolpyruvate
PFK1: Phosphofructokinase 1
PI: Proteasome inhibitor
PINK1: PTEN-induced kinase 1
PKB: Phosphokinase B
PKM2: Pyruvate kinase M2
POT1: Protection of telomeres 1
PP2A: Protein phosphatase 2A
PRAD: Prostate adenocarcinoma
p-RB: Phosphorylated RB protein
PR-DUB: Polycomb repressive deubiquitinase
PROTAC: Proteolysis targeting chimera
PRR: Pattern recognition receptor
PSMB: Proteasome beta
PSMD14: Proteasome non-ATPase regulatory subunit 14
RACGAP1: Rac GTPase-activating protein 1
RAP1: Ras-related protein 1
RB: Retinoblastoma
RBCK1: RANBP2-type and C3HC4-type zinc finger containing 1
RCC: Renal cell carcinoma
RFP2: Ret finger protein 2
RIPK1: Receptor-interacting protein kinase 1
RLIM: Ring finger LIM domain-interacting protein
RNF2: Ring finger protein 2
SAG: Sensitive to apoptosis gene
SAGA: Spt-Ada-Gcn5 acetyltransferase
SAMHD1: SAM and HD domain containing protein 1
SETDB1: SET domain bifurcated 1
SHARPIN: SHANK-associated RH domain-interacting protein
SIAH2: Seven in absentia homolog 2
SKP2: S-phase kinase-associated protein 2
SLC7A11: Solute carrier family seven member 11
SMAD7: Mothers against decapentaplegic homolog 7
SPOP: Speckle-type POZ protein
STD NMR: Saturation transfer differential nuclear magnetic resonance
STUB1: STIP1 homology and U-box-containing protein 1
SUMO: Small ubiquitin-related modifiers
SUZ12: Suppressor of zeste 12
SWI/SNF: Switch/sucrose non-fermentable
SYVN1: Synovial apoptosis inhibitor 1
TAB2: TAK1-binding protein 2
TAK1: Transforming growth factor ß-activated kinase 1
T-ALL: T-cell acute lymphoblastic leukemia
TBK1: TANK-binding kinase 1
TERT: Telomerase reverse transcriptase
TIN2: TRF-interacting nuclear protein 2
TIS: Tumor suppressor gene loss-induced senescence
TLR: Toll-like receptors
TME: Tumor microenvironment
TMM: Telomere DNA maintenance mechanism
TMUB1: Transmembrane ubiquitin-like domain 1
TMZ: Temozolomide
TNFAIP1: Tumor necrosis factor alpha-induced protein 1
TNFR2: TNF receptor 2
TPP1: Telomere protection protein 1
TRAF4: TNF receptor-associated factor 4
TRAP1: Tumor necrosis factor receptor-related protein 1
TRF1: Telomere repeat binding factor 1
TRIM7: Tripartite motif protein 7
TSGs: Tumor suppressor genes
U2AF65: U2 auxiliary factor 65
Ub: Ubiquitin
UBC13: Ubiquitin-conjugating enzyme 13
UBE2B: Ubiquitin-binding enzyme E2B
UBE2T: Ubiquitin-conjugating enzyme E2T
UBE3C: Ub-protein Ligase E3C
UBLs: Ubiquitin-like proteins
UBR5: Ubiquitin ligase E3 component N-recognition protein 5
UCHL5: Ubiquitin carboxyl-terminal hydrolase isozyme L5
UEV1A: Ubiquitin-like protein 1 activating enzyme
ULF1: Unexpected low fertilization
UPS: Ubiquitin–proteasome system
USP2: Ubiquitin-specific protease 2
VDAC1: Voltage-dependent anion channel 1
VEGF: Vascular endothelial growth factor
VHL: Von-hippel lindau
VSMC: Vascular smooth muscle cells
WWP2: WW domain containing E3 ubiquitin protein ligase 2
XIAP: X-linked apoptosis inhibitory protein
YTHDF2: YTH N6-methyladenosine RNA binding protein F2
ZMYM2: Zinc finger MYM-type protein 2
αKG: α-Ketoglutarate
β-TRCP: β-Transducin repeat-containing protein
γH2AX: Gamma-H2A histone family member X

## Glossary

### E3 ubiquitin ligases

E3 ubiquitin ligases: enzymes that facilitate the transfer of ubiquitin from a ubiquitin conjugating enzyme (E2) to a specific substrate protein, thereby marking it for degradation. E3 ubiquitin ligases are primarily classified into three groups based on their structural domains: RING finger ligases, HECT ligases, and RBR ligases.
Parkin: an E3 ubiquitin ligase from the RBR family, targets proteins for degradation. In cancer, Parkin ubiquitinates key proteins like p53, Cyclin E, HIF-1α, and PARIS, influencing cancer progression and tumor suppression.
F-box and WD repeat domain-containing 7 (FBXW7): a member of the F-box protein family, regulates cancer cell growth by ubiquitinating oncoproteins like c-Myc, Cyclin E, and Notch.
F-box protein 22 (FBXO22): an E3 ubiquitin ligase from the F-box protein family. It can ubiquitinate p21, LSD1, and ERα, leading to their degradation and influencing cell cycle progression, gene expression, and hormone receptor signaling.
Tripartite motif protein 7 (TRIM7): an E3 ubiquitin ligase from the TRIM family, targets proteins for degradation by ubiquitin-proteasome system. The target proteins of TRIM7 include β-catenin and p53.
Ring finger protein 2 (RNF2): an E3 ubiquitin ligase belonging to the RING finger protein family, impacts cancer progression by targeting proteins such as H2A and BMI1 for ubiquitination.
E3 ligase complex Cul4-DDB1-CRBN-RBX1 (CRL4^CRBN^): an E3 ubiquitin ligase from the Cullin-RING family, regulates cancer cell growth by ubiquitinating target proteins like IKZF1 and IKZF3.
Linear ubiquitin chain assembly complex (LUBAC): an E3 ubiquitin ligase complex composed of HOIP, HOIL-1, and SHARPIN that regulates signaling pathways and cellular processes by adding linear ubiquitin chains to specific target proteins. In cancer, its substrates include NEMO, CYLD, TNFR1, RIPK1, and MLKL, which play crucial roles in the NF-κB signaling pathway.
Synovial apoptosis inhibitor 1 (SYVN1): is an E3 ubiquitin ligase that plays a key role in the endoplasmic reticulum-associated degradation (ERAD) pathway. It targets misfolded proteins for ubiquitination and subsequent degradation. In cancer, its substrates include p53, IRE1α, MCL1, and HIF-1α.
HECT domain and RCC-1-like domain one gene (HERC1): a ubiquitin ligase containing HECT and RCC1-like domains, is involved in various cellular processes such as protein degradation, cell cycle regulation, and DNA damage response, by regulating the stability and function of HSP70 and Caveolin-1. Abnormal HERC1 function may lead to uncontrolled growth and anti-apoptosis of cancer cells.
Neural precursor cells expressed developmentally downregulated 8 (NEDD8): a small ubiquitin-like protein that regulates protein modification processes by neddylating target proteins, impacting crucial cellular processes such as cell cycle regulation, DNA repair, protein degradation, and signal transduction. In cancer, NEDD8 modifies substrates like cullin, which are part of the E3 ubiquitin ligase complex SKP1-CUL1-F-box protein (SCF).
Breast cancer susceptibility gene 1 (BRCA1): a tumor suppressor gene that plays a pivotal role in DNA repair, cell cycle regulation, and apoptosis. Mutations in BRCA1 significantly increase the risk of breast, ovarian, and other cancers, making it a biomarker for hereditary cancer susceptibility and guiding tailored treatment approaches. BRCA1’s substrates include proteins involved in DNA repair pathways, such as RAD51, and cell cycle regulators like p21 and cyclin D1.
F-box and leucine-rich repeat protein 18 (Fbxl18): as a member of the F-box protein family, FBXL18 plays a significant role in cancer by participating in the Skp, Cullin, and F-box containing (SCF) E3 ubiquitin ligase complex. In cancer, FBXL18 has been implicated in targeting substrates like cyclin D1, a key regulator of cell cycle progression, and c-Myc, an oncogenic transcription factor, thus affecting tumor growth and progression.
Speckle-type POZ protein (SPOP): an adaptor protein for the Cullin3-RING E3 ubiquitin ligase complex, SPOP plays a crucial role in cancer. It regulates protein degradation by targeting specific substrates for ubiquitination. In cancer, SPOP has been implicated in the degradation of substrates such as AR and ERG, impacting oncogenic signaling pathways and contributing to tumor development and progression.
Ubiquitin-conjugating enzyme 13 (UBC13): an E2 ubiquitin conjugating enzyme that forms K63-linked polyubiquitin chains. In cancer, UBC13 is involved in the regulation of substrates such as TRAF6 and RIPK1, which play key roles in NF-κB signaling and cell survival.
Ubiquitin-like protein 1 activating enzyme (UEV1A): a co-factor for UBC13, plays a crucial role in cancer by facilitating K63-linked polyubiquitination of proteins such as BRCA1 and FANCD2 for DNA repair, IκB kinase for NF-κB signaling, and cyclin-dependent kinase inhibitors like p21 and p27 for cell cycle regulation.
Trib1-COP1 complex: an instrumental player in controlling cell proliferation, differentiation, and metabolism, the Trib1-COP1 complex significantly impacts cancer development by ubiquitinating and regulating the stability of proteins such as c-Jun, p53, and AKT.

### Deubiquitinase (DUB)s

DUB: an enzyme that removes ubiquitin molecules from proteins in a process called deubiquitination, which counteracts the effects of ubiquitination by preventing protein degradation and regulating protein function and localization.
OTU domain-containing ubiquitin aldehyde-binding protein 2 (OTUB2): an OTU domain deubiquitinating enzyme, regulates NF-κB signaling by deubiquitinating key components such as IKKγ and RIP1. This promotes inflammation and potentially acts as a tumor suppressor in cancer.
OTU deubiquitinase with linear linkage specificity (OTULIN): a deubiquitinase regulates the NF-κB signaling pathway by specifically removing linear ubiquitin chains, potentially affecting the stability of IKKγ and NEMO, thus influencing inflammatory responses and immune regulation in cancer.
COP9 signalosome subunit 5 (CSN5): a part of the COP9 signalosome complex, promotes the degradation of key proteins such as p27, c-Myc, and HIF-1α in cancer, enhancing cell cycle progression, cell growth, and angiogenesis.
Cezanne-1: an important deubiquitinating enzyme, modulates protein stability and activity in cancer. Its target proteins include IKKα/β, TRAF2, and SMAD7.

### Key Concepts

Ubiquitin-specific protease (USP): is a large family of enzymes that plays a critical role in deubiquitination, which is the removal of ubiquitin molecules from protein substrates. USPs are involved in various pathologies due to their central role in regulating protein stability and function. In cancer, for example, alterations in the activity or expression of certain USPs can lead to the dysregulation of oncogenes or tumor suppressors, contributing to cancer progression.
Mitochondrial outer membrane permeabilization (MOMP): a process involving the penetration of the mitochondrial outer membrane, usually triggered by apoptotic signals.
26S proteasome: comprising a 20S core particle and two 19S regulatory particles, and functions as a protein degradation system within cells. It is crucial for maintaining cellular homeostasis by degrading unwanted proteins.
Cancer-Associated Fibroblasts (CAFs): a subtype of fibroblast cells in the tumor microenvironment, significantly impact tumor growth, invasion, and metastasis by interacting with tumor cells through the secretion of growth factors, extracellular matrix proteins, and inflammatory mediators.
Tumor Microenvironment (TME): a critical component of tumor biology. TME encompasses blood vessels, immune cells, fibroblasts, and extracellular matrix, playing vital roles in tumor growth, progression, angiogenesis, immune evasion, and metastasis.
Epithelial-Mesenchymal Transition (EMT): an essential process in cancer, enhances tumor invasiveness and metastasis by causing the loss of epithelial markers like E-cadherin and the gain of mesenchymal markers such as N-cadherin, vimentin, and fibronectin. This process is regulated by transcription factors like Snail, Slug, Twist, and ZEB1/2, and driven by signaling pathways including TGF-β, Wnt/β-catenin, Notch, and Hedgehog.
Dedifferentiation: a process in which mature, specialized cells revert to a more primitive, unspecialized state, often regaining the ability to divide and differentiate into other cell types.
Transdifferentiation: a direct conversion of one differentiated cell type into another without reverting to a stem cell state.
Proteolysis targeting chimeras (PROTACs): are bifunctional small molecules consisting of two specific ligands: one for an E3 ubiquitin ligase and another that binds to a target protein. These ligands are connected by a linker, creating a trimeric complex-target protein ligand-Linker-E3 ligand. The E3 ligase tags the target protein with ubiquitin, leading to its specific degradation through the ubiquitin-proteasome pathway.
Molecular glue: is used in the field of drug discovery and biochemistry to refer to a small molecule that promotes the interaction between two proteins, typically leading to a therapeutic effect. These molecules act by “gluing” a target protein and a ubiquitin ligase together, facilitating the ubiquitination and subsequent degradation of the target protein by the proteasome system.
